# Antioxidant Activity and Cytotoxicity Evaluation of New Catechol Hydrazinyl-Thiazole Derivatives as Potential Protectors in Retinal Degenerative Processes

**DOI:** 10.3390/antiox14060646

**Published:** 2025-05-28

**Authors:** Răzvan-Geo Antemie, Gabriel Marc, Raluca Pele, Ionel Fizeșan, Ionuț-Valentin Creștin, Raluca Borlan, Panagiotis Theodosis-Nobelos, Eleni A. Rekka, Ovidiu Oniga, Ovidiu Crișan, Adrian Pîrnău, Laurian Vlase, Simona Valeria Clichici

**Affiliations:** 1 Department of Physiology, Faculty of Medicine, “Iuliu Haţieganu” University of Medicine and Pharmacy, 400006 Cluj-Napoca, Romania; antemie_razvan_geo@elearn.umfcluj.ro (R.-G.A.); sclichici@umfcluj.ro (S.V.C.); 2 Department of Organic Chemistry, “Iuliu Hațieganu” University of Medicine and Pharmacy, 41 Victor Babeș Street, 400012 Cluj-Napoca, Romania; ocrisan@umfcluj.ro; 3 Department of Pharmaceutical Chemistry, “Iuliu Hațieganu” University of Medicine and Pharmacy, 41 Victor Babeș Street, 400012 Cluj-Napoca, Romania; raluca.pele@umfcluj.ro (R.P.); ooniga@umfcluj.ro (O.O.); 4 Department of Toxicology, Faculty of Pharmacy, “Iuliu Hațieganu” University of Medicine and Pharmacy, 8 Victor Babeș, 400012 Cluj-Napoca, Romania; ionel.fizesan@umfcluj.ro (I.F.); ionut.vale.crestin@elearn.umfcluj.ro (I.-V.C.); 5 Nanobiophotonics and Laser Microspectroscopy Centre, Interdisciplinary Research Institute in Bio-Nano-Sciences, Babeș-Bolyai University, 400084 Cluj-Napoca, Romania; raluca.borlan@ubbcluj.ro; 6 Department of Pharmacy, School of Health Sciences, Frederick University, Nicosia 1036, Cyprus; hsc.np@frederick.ac.cy; 7 Department of Pharmaceutical Chemistry, School of Pharmacy, Aristotelian University of Thessaloniki, 54124 Thessaloniki, Greece; rekka@pharm.auth.gr; 8 National Institute for Research and Development of Isotopic and Molecular Technologies, 67-103 Donath Street, 400293 Cluj-Napoca, Romania; apirnau@itim-cj.ro; 9 Department of Pharmaceutical Technology and Biopharmaceutics, “Iuliu Hațieganu” University of Medicine and Pharmacy, 41 Victor Babeș Street, 400012 Cluj-Napoca, Romania; laurian.vlase@umfcluj.ro

**Keywords:** antioxidant, antiradical, thiazole, polyphenol, retina, age-related macular degeneration, ARPE-19 cell line

## Abstract

Retinal degenerative processes such as age-related macular degeneration are at the center of many ongoing research studies, as their impact on the general population is significant, with severe visual impairment and even irreversible vision loss if left untreated. Currently, there are few efficient treatments available to stop or limit its progression. In the present paper, a molecular hybridization approach was employed to develop novel compounds that address this issue. By adding either 2-butenal or a β-ionone-derived residue to the hydrazone-catechol-thiazole scaffold, two compounds were designed and synthesized: **5a** and **5b**. After being characterized by mass spectrometry and nuclear magnetic resonance, and proving potent antioxidant activity in the in vitro assays, the cytotoxicity evaluation using the ARPE-19, BJ, and A549 cell lines revealed a surprisingly low-dose effect of **5a** and the unexpected cytotoxic activity of **5b**, despite its β-ionone moiety, known for its significant therapeutic properties.

## 1. Introduction

As human life expectancy increases, it inevitably leads to an increase in age-related pathologies such as cancer, cardiovascular, and ocular diseases. Retinal degenerative diseases encompass a complex group of disorders that are responsible for vision loss in numerous patients. Age-related macular degeneration (AMD) is one of the leading causes of blindness in elderly people [1,2]. It is characterized by the accumulation of lipofuscin and *N*-retinylidene-*N*-retinylethanolamine (A2E) in the retinal pigment epithelium (RPE) cells, a monolayer of pigmented and polarized cells. This accumulation is a result of an incomplete digestion of photoreceptor outer segments [3], which causes RPE dysfunction and ultimately leads to photoreceptor death and irreversible vision loss. The roles of the RPE include supplying vital nutrients to the photoreceptor layer, phagocytosis of used photoreceptor outer segments, and the transportation of metabolites and fluids, as it is part of the outer blood-retinal barrier [4]. The accumulation of lipofuscin and A2E determines significant oxidative cellular stress, especially when exposed to blue light, that invariably leads to apoptosis, necroptosis, ferroptosis, and autophagy-related cell death, if left unaddressed [5,6].

AMD is classified into dry (without neovascularization, also referred to as atrophic or non-exudative) and wet (with neovascularization, also known as exudative). While the latter benefits from treatment with intraocular injections of anti-VEGF antibodies, the same cannot be said about the former. There is limited treatment that can inhibit dry AMD [7] or limit its progression to the wet form [8]. In the United States alone, 1 million patients are affected by the late stage of dry AMD, with one out of four being legally blind [9,10]. In Europe, the prevalence of AMD was 16.2% among older people (aged 70 years and older), based on the Rotterdam classification, and it is expected to double by 2040 [11]. AMD is a complex multifactorial process, which is closely linked to increased oxidative stress and chronic inflammation in the retina and the surrounding tissues, leading to progressive damage of the photoreceptor cells and the RPE, ultimately contributing to vision loss [12,13].

**Figure 1:**
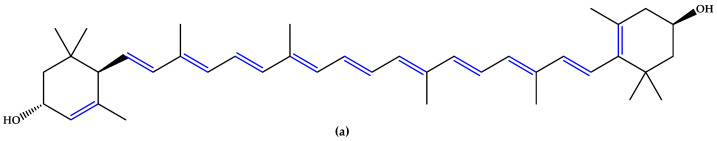

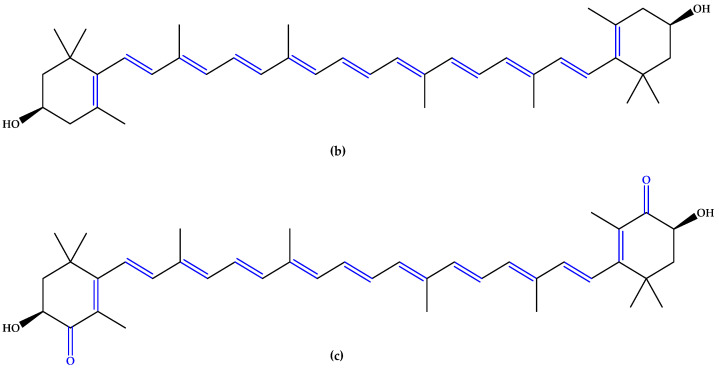
(**a**) Lutein. (**b**) Zeaxanthin. (**c**) Astaxanthin. Unsaturated bonds are depicted in blue.

For preventive intervention, supplements with antioxidants and minerals are globally consumed [14], with lutein and zeaxanthin (Figure 1), two carotenoid compounds and physiological macular pigments, viewed as preventive options and included in the dietary approach [15]. Chemically, lutein is formed from the C40 isoprenoid carotenoids` characteristic structure, having 10 conjugated double bonds (9 in the polyene chain and 1 in the β-ionone ring). Comparatively, zeaxanthin contains 11 conjugated double bonds (9 in the polyene chain and 2 in the β-ionone rings) [16]. Other unsaturated compounds with multiple conjugated double bonds, such as astaxanthin (Figure 1), fucoxanthin, and crocin, also suppress reactive oxygen species (ROS) generation and inflammation due to a region of decentralized electrons that results from the presence of the respective double bonds alternating with simple bonds or carbonyl groups. Particularly, astaxanthin contains 11 conjugated double bonds (9 in the polyene chain and 2 in the β-ionone-4,4′-dione rings) [17,18,19,20,21].

**Figure 2:**
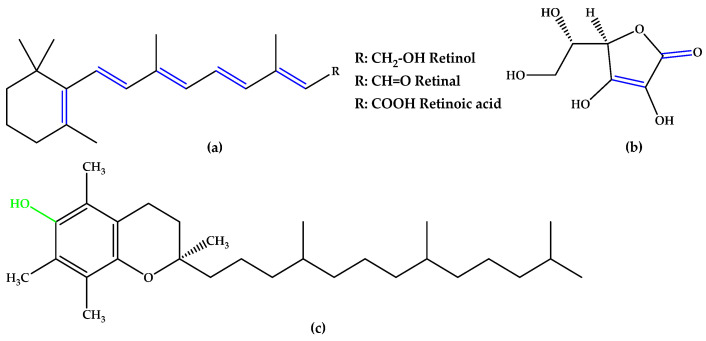
(**a**) Vitamins A. (**b**) Vitamin C (ascorbic acid). (**c**) Vitamin E (α-tocopherol). Unsaturated bonds are depicted in blue and phenols in green.

In the same trend of antioxidant supplementation, vitamins A, C, and E (Figure 2), known to possess antioxidant activity, were extensively studied for quenching ROS in retinal tissue through non-enzymatic reactions. The alternation of double bond-simple bond or double bond-carbonyl group, which is involved in ROS suppression, was observed in vitamins A and C. Tocopherol contains a phenolic OH group that can scavenge ROS [12].

**Figure 3:**
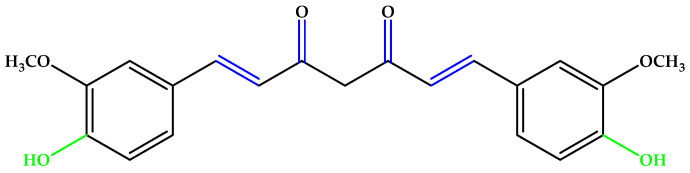
Curcumin. Unsaturated bonds are depicted in blue and phenols in green.

Curcumin (Figure 3), an unsaturated polyphenol from turmeric (*Curcuma longa* Linn.) with its metabolite hexahydrocurcumin, showed a cellular protective effect against blue light-induced phototoxicity in RPE cells. It is formed from two phenolic rings holding *ortho*-methoxy groups, linked by an α,β-unsaturated seven-carbon β-diketone moiety [22,23].

**Figure 4:**
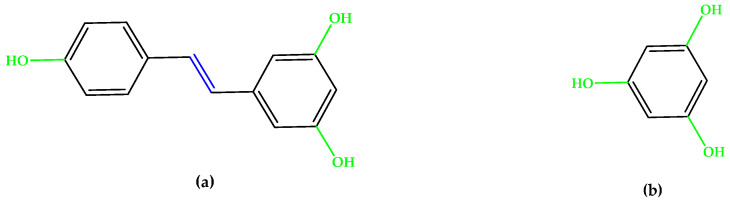
(**a**) Resveratrol. (**b**) Phloroglucinol. Unsaturated bonds are depicted in blue and phenols in green.

Resveratrol (Figure 4), a triphenol stilbenoid (two phenyl rings linked through an ethylene bridge), preserved the cytoskeleton architecture of the RPE cells by ameliorating the redox balance and the mitochondrial integrity [24,25]. Other phenols and polyphenols, such as epicatechin, piceid octanoate, puerarin, oleuropein, or phloroglucinol (Figure 4) were also studied, and numerous results indicate that antioxidant molecules may inhibit the onset and progression of AMD [13,26,27,28].

**Figure 5:**
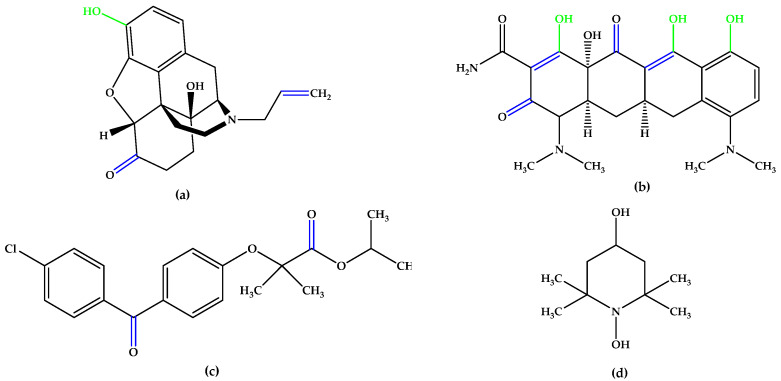
(**a**) Naloxone. (**b**) Minocycline. (**c**) Fenofibrate. (**d**) OT-674. Unsaturated bonds are depicted in blue, and phenols and enols in green.

The synthetic experimental compound OT-674 (Figure 5) suppressed the photooxidative processes initiated by the fluorophore A2E [29], while authorized repurposed drugs, such as naloxone (with a phenolic group at the C3 position and double bonds in the izochinolinphenantrenic ring), minocycline (containing three phenolic groups attached on the tetracyclic naphthacene carboxamide ring system and a characteristic arrangement of double bonds), and fenofibrate, a chlorobenzophenone derivative (Figure 5), were evaluated under oxidative stress conditions in experimental models mimicking dry AMD pathogenesis and have shown encouraging results [30,31,32,33,34,35].

New interventions for retinal degenerative diseases have thus become necessary, and many research groups are searching for potential candidates to prevent AMD in either natural or synthetic sources. Encouraged by the reports in the literature on antioxidants and drug design, we designed new synthetic antioxidants with potential use in AMD prevention or treatment. Over the years, azoles have played a key role in the design and synthesis of compounds with significant biological activity [36,37,38,39], with various drugs incorporating the thiazole nucleus, exhibiting promising bioactivities, and demonstrating great potential in medicine. Hydrazinyl thiazoles were reported to have antioxidant activity per se, which was attributed to this moiety, all the other functional groups in the respective molecules being inert in terms of antioxidant activity [40].

Compounds bearing phenol moieties have been reported to exhibit antioxidant activity [41,42,43], which is enhanced by the *ortho* positioning of the phenol OH group, known as catechol. Compared to other relative positioning of two phenol groups, this arrangement demonstrates greater activity than resorcinol or hydroquinone derivatives [44,45,46,47] and was considered advantageous in the development of the new compounds reported in this paper.

**Figure 6:**
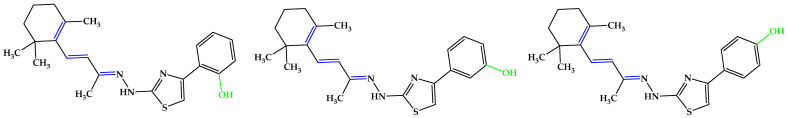
Compounds reported by Huang et al. [75], which exhibited the highest antioxidant activity in the in vitro assays, analogs with **5b**. Unsaturated bonds are depicted in blue and phenols in green.

The main carotenoids in the human body are α-carotene, β-carotene, β-cryptoxanthin, lycopene, and lutein [48]. The cleavage of β-carotene by the inner mitochondrial β-carotene oxygenase 2 (BCO2) enzyme yields two β-ionone moieties and one rosafluene [49,50,51,52]. Ionones, which are naturally occurring compounds found in flowers, fruits, and vegetables, such as carrots, tomatoes, melons, raspberries, apricots, plums, apples, and figs [53,54,55,56,57,58] are secondary plant metabolism products that share mevalonic acid as a common precursor [59,60]. It has also been found in cow’s milk after alfalfa pasture [61,62]. The β-isomer of ionone has an impressive array of biological effects mediated by the olfactory receptor family subunit E member 2 (OR51E2), ranging from anticancer and melanogenesis to anti-inflammatory and antimicrobial properties [63,64,65], and even regulating the activity of cell cycle regulatory proteins [60,66,67,68,69,70], pro-apoptotic and anti-apoptotic proteins [60,62,67,70,71,72,73], and pro-inflammatory mediators [67,74]. In recent years, it has received increased attention from the medical and pharmaceutical communities for its potential health benefits in humans, being used as a scaffold for the design of new drugs. Huang et al. [75] reported the synthesis of several β-ionone 4-phenyl-thiazolyl hydrazone derivatives with significant antiradical activity in the ABTS^•+^ scavenging assay, which was deemed a strong starting point for the current research. The most active compounds were those possessing a phenolic OH group on the phenyl ring, compared to the others from their respective series (Figure 6).

**Figure 7:**
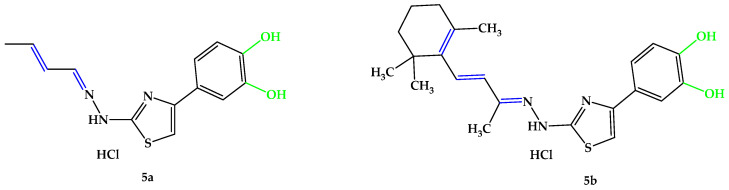
Compounds **5a**,**b** developed in the current research. Unsaturated bonds are depicted in blue and phenols in green.

An interesting viewpoint for the present research emerged after studying these significant functional groups and structural particularities that exhibit antioxidant activity. According to this research perspective, the hydrazone-catechol-thiazole scaffold represents a complex pharmacophore with broad biological activities [76,77,78,79,80,81,82]. Given the relevant findings, along with our group’s previous results in the field of catechol-derived thiazoles and the intrinsic activity of the thiazolyl-hydrazone derivatives with no additional antioxidant moieties [40], we were motivated to synthesize and evaluate the diphenol compound **5b** reported in the current paper, expecting it to exhibit greater activity than those from Huang et al.’s study, which featured monophenols [75]. In the present research, we developed two structurally analogous compounds (Figure 7) with strong antioxidant activity to address the ongoing problem of oxidative stress, a recognized and significant contributing factor in the pathogenesis of retinal diseases, such as AMD. In the first part of our study, utilizing a molecular hybridization approach where active pharmacophoric units were linked in a single matrix, we aimed to create new molecular hybrids based on the hydrazone-catechol-thiazole scaffold with conjugated unsaturated substituents on the azomethine group. One of the compounds was designed to have a lower molecular weight with a linear aliphatic unsaturated conjugated substituent using 2-butenal, while the other was substituted with an aliphatic unsaturated conjugated residue derived from β-ionone, displaying a closer similarity to vitamin A. Both **5a** and **5b** were designed to incorporate the conjugated unsaturated system in position 2 of the central thiazole ring to form the conjugated π system typically found in the polyene chain of carotenoids.

As far as pharmaceutical chemistry is concerned, significant differences are expected to be identified between the two compounds in terms of reactivity, cellular penetrability, and toxicity due to the different substituents. We have assessed the chemical differences regarding chemical reactivity, fine electronic differences, and cellular tolerability on human retinal pigment epithelial cells (ARPE-19) as well as on two other cell types—normal human foreskin fibroblasts (BJ) and human lung adenocarcinoma cells (A549) for potential systemic implications. This study forms part of a broader research initiative to develop and evaluate therapeutic strategies for AMD [83].

## 2. Materials and Methods

### 2.1. Chemical Synthesis

All chemicals utilized in the current chemical synthesis were procured from local suppliers and were produced by Merck KGaA (Darmstadt, Germany). The purity of the intermediate compounds **3a**,**b** and the final compounds **5a**,**b** was initially assessed through thin-layer chromatography (TLC), followed by confirmation using RP-HPLC on an Agilent 1100 device (Agilent Technologies, Santa Clara, CA, USA). The solid powders of the synthesized compounds underwent melting point determination using an MPM-H1 apparatus (Schorpp Gerätetechnik, Überlingen, Germany) within glass capillaries.

Mass spectra were captured on the Agilent 1100 device employing the positive ionization mode for the intermediate **3a**,**b** compounds and negative ionization mode for the final compounds **5a**,**b** on an Agilent Ion Trap SL mass spectrometer. The nuclear magnetic resonance (NMR) spectra of the tested compounds were obtained on an Avance NMR apparatus (Bruker, Karlsruhe, Germany) in dimethylsulfoxide-*d*_6_ (DMSO-*d*_6_), and chemical shift values were reported in δ units. Calibration of the device was performed using tetramethylsilane (TMS) and the signals were referenced to the residual solvent’s peak.

**Figure 8:**
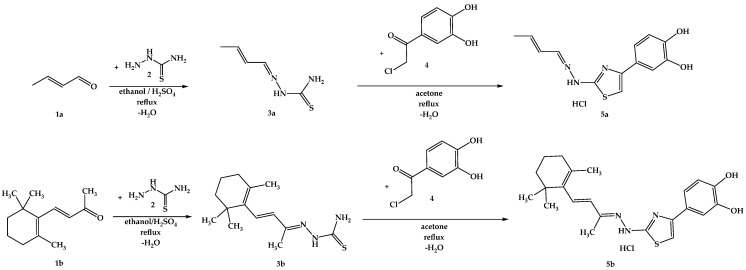
The chemical synthesis pathway used for the obtention of final thiazoles **5a**,**b**.

The synthesis pathway for obtaining the intermediate compounds **3a**,**b** and the final compounds **5a**,**b** is illustrated in Figure 8, following an adaptation of previously reported protocols [75,84,85,86]. Briefly, 20 mmol (1.82 g) of thiosemicarbazide (**2**) were dissolved in the minimum amount of boiling ethanol and a catalytic amount of 96% sulfuric acid was added. After, 20.5 mmol of the carbonylic compounds **1a** or **1b** were added dropwise to the solution. The mixtures were refluxed until the completion of the reaction was confirmed by TLC. The resulting precipitates were filtered under vacuum and crystallized from fresh ethanol, after which 6 mmol of thiosemicarbazones **3a**,**b** were dissolved in the minimum amount of boiling acetone, and 6.05 mmol (1.12 g) of 4-(chloroacetyl)catechol (**4**) were added. The mixtures were refluxed until the completion of the reaction, which was confirmed by TLC. Upon cooling, the resulting precipitates were filtered under vacuum and crystallized from fresh acetone. The resulting solid powders of compounds **5a**,**b** were immediately closed in storage tubes to prevent degradation.

### 2.2. In Vitro Antioxidant Evaluation

Absorption spectra of the tested compounds, ranging from 400 nm to 800 nm, were recorded using a Specord 210 PLUS UV-VIS spectrophotometer (Analytik Jena AG, Jena, Germany) using low-volume single-use 10 mm cuvettes and showed no absorption peaks near the wavelengths used in the assays. In all assays performed, the reference antioxidants were evaluated in the same way as the tested compounds to give a clear image of the antioxidant power of the tested compounds.

#### 2.2.1. Radical Scavenging Assays

The DPPH^•^ radical scavenging assay is based on transferring a hydrogen atom from the tested compounds to the violet stable free radical DPPH^•^ (2,2-diphenyl-1-picrylhydrazyl radical), converting it into a yellow compound. The assay was conducted using tested compounds and references in concentrations ranging from 2 to 50 µM, according to our adaptation of the protocol of Brand-Williams et al. [87,88]. The absorbance of the samples is given by the remaining unreacted reagent radical, and it is inversely proportional to the amount of DPPH^•^ neutralized. The absorbance of the samples was measured at λ = 517 nm after 30 min of incubation in the dark at room temperature [87,88]. The DPPH^•^ radical scavenging activity of the compounds was calculated using Equation (1):

(1) $${DPPH}^{•}\;scavenging\;(\%)=\frac{control\;absorbance-sample\;absorbance}{control\;absorbance}\times 100$$

The ABTS^•+^ (2,2′-azinobis-(3-ethylbenzothiazoline-6-sulfonic acid) decolorization assay, based on the method of Re et al., was performed in the concentration range of 1 to 25 µM according to protocols previously reported by our group. The reagent was prepared in a 0.1 M potassium phosphate buffer (pH 7.4) and activated overnight with MnO_2_ [89]. Prior to use, the stability of the ABTS^•+^ reagent was verified by checking its absorbance at λ = 734 nm over one hour, ensuring a consistent absorption of around 0.7. The mixtures were incubated for 10 min in the dark at room temperature. The absorbance of the samples is given by the remaining unreacted reagent radical, and it is inversely proportional to the amount of ABTS^•+^ neutralized. The ABTS^•+^ scavenging activity of compounds was calculated using Equation (2).

(2) $${{ABTS}^{•}}^{+}\;scavenging\;(\%)=\frac{control\;absorbance-sample\;absorbance}{control\;absorbance}\times 100$$

The ^1^O_2_ scavenging assay was employed using a previously established indirect protocol from our research group [90]. In brief, indocyanine green (ICG), from Sigma–Aldrich (St. Louis, MO, USA), was utilized to induce ^1^O_2_ production in the presence of **5a** and **5b**. Ascorbic acid was used as a reference antioxidant agent, and a blank sample without ^1^O_2_ scavengers was prepared. The reaction mixtures were exposed to a Micro Raman System R-3000 (Photonitech, Singapore, Singapore) laser diode,(λ = 785 nm, 190 mW power) for a total time of 100 s. To evaluate the ^1^O_2_ scavenging activity of the compounds **5a**,**b**, the degradation of 1,3-diphenylisobenzofuran (DPBF), obtained from Alfa Aesar (Haverhill, MA, USA), was measured using a V-730 spectrophotometer (Jasco International Co., Ltd., Tokyo, Japan). To determine the ^1^O_2_ quantum yields (Φ(^1^O_2_)) of ICG in the presence of the studied molecules, the following equation was used: Φ(^1^O_2_) = Φ(^1^O_2_) ^ICG^ × ((S × F^ICG^)/(S^ICG^ × F)), where S represents the slope of the absorbance difference of DPBF over time, F is the absorption correction factor (F = 1 − 10^−OD^), and OD is the optical density at the excitation wavelength, λ = 785 nm. The Φ(^1^O_2_) equal to 15.0% of free ICG was used as a reference [91]. The concentrations utilized in the experiment were 0.1 mM for DPBF and 10^−5^ M for ICG and the ^1^O_2_ scavengers. Free DPBF was used as a control.

#### 2.2.2. Electron Transfer Assays

To evaluate the capacity of compounds **5a**,**b** to reduce different metal-based oxidizing agents to lower oxidation states in various environments, three assays were performed. The results of the evaluation of the electron ability transfer of the tested compound were expressed as molar equivalents of the reference compound per one mol of the tested compound using the formula:

$$\%\;of\;control\;activity=\frac{sample\;absorbance}{reference\;absorbance}\times 100$$

The Total Antioxidant Capacity (TAC) assay operates on the principle of reducing molybdenum (VI) to molybdenum (V) by the antioxidants being assessed, resulting in the formation of a blue-green complex. This assay was performed following the methodology detailed in our previous publication, which was based on initial reports from the literature [92,93]. A volume of 10 mL of the reagent (0.6 M sulfuric acid, 28 mM sodium phosphate, and 4 mM ammonium molybdate) and 1000 µL of the compound or standard solution from 2 mM stock solutions were mixed in sealed glass test tubes. These were then incubated in a water bath at 95 °C for 90 min. After cooling to room temperature, 1 mL of each solution was diluted with 1 mL of water. The absorbance of the solutions was subsequently measured at λ = 695 nm.

In the Reducing Power (RP) assay, the tested compounds reduce ferric ions from potassium ferricyanide, forming ferrocyanide, which produces a blue complex. The protocol used is an adaptation of previously reported methodologies [92]. In glass test tubes, 1000 µL of a 0.2 mM compound or standard stock solution were mixed with 400 µL of phosphate buffer (0.2 M, pH 6.6) and 400 µL of K_3_[Fe(CN)_6_] solution (1% *w*/*v*). The tubes were sealed and incubated for 20 min in a water bath at 50 °C. After cooling to room temperature, 500 µL of trichloroacetic acid (10% *w*/*w*) was added to all test tubes, and the mixtures were allowed to equilibrate to room temperature for 30 min. Subsequently, 250 µL of the resulting solutions were mixed with 140 µL of FeCl_3_ solution (0.1% *w*/*v*) and 1000 µL of distilled water, and the absorbance of the resulting solutions was measured at λ = 695 nm.

The Ferric Reducing Antioxidant Potential (FRAP) assay measures the ability of an antioxidant to transfer an electron to Fe^3+^, converting it to Fe^2+^. The resulting Fe^2+^ ions are then chelated by 2,4,6-tripyridyl-s-triazine, giving a strong blue complex [94]. According to the reports [88,94,95] 500 µL of the compound or reference compound solution (0.2 mM) were mixed with 600 µL of FRAP reagent and 1200 µL of acetate buffer (0.3 M, pH 3.6). The solutions were thoroughly mixed in the dark, and their absorbance was measured spectrophotometrically at λ = 593 nm.

#### 2.2.3. Ferrous Ions Chelation Assay

To assess the ferrous ion-chelating activity of compounds **5a**,**b**, an assay based on the method originally described by Dinis et al. was employed [92,96,97,98,99,100]. Solutions of the tested compounds or ethylenediaminetetraacetic acid disodium salt (EDTA-Na_2_), used as a positive control, were thoroughly mixed with 0.5 mL of FeSO_4_ (0.125 mM) and 0.5 mL of ferrozine (0.315 mM). After a 10-min incubation in the dark at room temperature, the absorbance was measured at λ = 562 nm.

#### 2.2.4. Lipid Peroxidation Inhibition Assay (LPI)

Rat liver microsomes were homogenized in saline, and the resulting homogenates were centrifuged at 9000× *g* for 20 min at 4 °C to remove cell debris. The supernatant underwent ultracentrifugation at 110,000× *g* for 40 min at 4 °C. The pelleted microsomal fraction was subsequently washed with saline via another round of ultracentrifugation under identical conditions. Thereafter, the pellet was resuspended in Tris-HCl buffer (pH 7.4) to achieve a concentration equivalent to 0.5 g liver per mL and was stored until further analysis (at −80 °C). For the assay, the microsomal preparation was thermally inactivated by incubation at 90 °C for 90 s, then homogenized again and diluted to a final concentration equivalent to 4 mM fatty acid equivalents.

The reaction mixtures for lipid peroxidation included the heat-inactivated microsomal fraction, the test compounds dissolved in dimethyl sulfoxide (DMSO) at final concentrations ranging from 1 μM to 1 mM (or DMSO alone for the control group), and an ascorbic acid/Fe^2+^ system. All mixtures were incubated at 37 °C under air with continuous shaking. Aliquots (0.3 mL) were taken over a total incubation period of 45 min and mixed with 2 mL of an ice-cold quenching solution composed of 2-thiobarbituric acid, trichloroacetic acid, hydrochloric acid, and butylated hydroxytoluene to terminate the peroxidation reaction. This was followed by heating at 90 °C for 20 min and centrifugation at 3000 rpm for 15 min. As a result of the breakdown of unsaturated fatty acids, malondialdehyde reacts with the 2-thiobarbituric acid to produce a pink compound due to the formation of a Schiff base.

The extent of lipid peroxidation was measured spectrophotometrically at 535/600 nm, which is proportional to the amount of the chromogen reaction product formed between 2-thiobarbituric acid and malondialdehyde. The determinations were performed in triplicate, and the standard deviation was within ±10% of the mean value [101,102].

### 2.3. In Silico Studies

#### 2.3.1. Quantum and Thermodynamic Calculations

To describe the antioxidant potential of polyphenols, theoretical methods predicting their molecular behavior were employed. In this study, we investigated the impact of the compounds’ structure on their antioxidant properties. We computed theoretical quantum parameters, including the Highest Occupied Molecular Orbital (HOMO) and Lowest Unoccupied Molecular Orbital (LUMO), for all compounds. Additionally, Bond Dissociation Enthalpy (BDE) calculations for the phenol groups and the hydrazones were conducted. The analysis aims to provide insights into the in vitro antioxidant assay results concerning the compounds’ structure.

The HOMO-LUMO gap offers insights into the electron-donating and accepting capabilities of antioxidants, providing a glimpse into their redox properties. Additionally, BDE analysis focuses on the strength of bonds related to hydroxyl groups, a crucial factor determining the compounds’ ability to donate hydrogen atoms and neutralize free radicals.

Literature suggests that the hydrogen atom transfer (HAT) mechanism plays a crucial role when polyphenols act as antioxidants. This mechanism involves the homolytic break of the O-H bond, accompanied by the simultaneous release of a hydrogen atom (comprising a proton and an electron). Consequently, our current research extensively explores this mechanism, with its schematization represented by the chemical reaction Ar-OH → Ar-O^•^ + H^•^.

The ease with which the O-H bond breaks and releases H^•^ is closely tied to the antiradical properties of the compounds and their ability to conjugate the resulting odd electron from the phenoxy radical across the molecule to achieve a low-energy state. In contrast, mechanisms such as single electron transfer-proton transfer (SET-PT) and sequential proton loss electron transfer (SPLET) are less favorable for phenolic compounds and were not assessed in our present research.

Computations were carried out using Spartan24 (Wavefunction, CA, USA) at the B3LYP level of theory with the 6-311+G(d,p) basis set for compounds **5a**,**b** in vacuum, nonpolar solvent (ε = 7.43), and water. This approach provides insights into how the solvent’s polarity influences the antioxidant activity of the compounds [88,103,104,105].

#### 2.3.2. ADME Study

In addition to a complex profile of compounds **5a**,**b**, computational studies were conducted. The aim was to predict molecular properties by using the SwissADME online server [106]. By drawing the chemical structure of each compound, a SMILES structure was generated and used in the study. The following parameters were determined and analyzed: molecular weight (Mw), number of rotatable bonds (Nrotbs), number of hydrogen-bond acceptors (HBAs), number of hydrogen-bond donors (HBDs), topological polar surface area (TPSA), logarithm of octanol/water partition coefficient (logP), water solubility (logS), gastrointestinal absorption (GI abs), brain-blood barrier (BBB) permeability, P-gp (P-glycoprotein) substrate, CYP450 inhibitors (CYP1A2, CYP2C19, CYP2C9, CYP2D6, CYP3A4), and Lipinski’s rule of five [107].

### 2.4. In Vitro Cytotoxicity Evaluation

#### 2.4.1. Cell Cultures

The ARPE-19 cell line was purchased from CLS Cell Lines Service GmbH (Eppelheim, Germany) and cultured in Dulbecco’s modified Eagle’s medium/F-12 (HAM) 1:1 (Biological Industries, Beit, Israel). The medium was supplemented with 10% fetal bovine serum (FBS) (Thermo Fisher Scientific, Waltham, MA, USA) and 1% penicillin-streptomycin (Biosera, Cholet, France). The cancerous cell line A549 was purchased from ATCC (Manassas, VA, USA) and cultivated in high-glucose Dulbecco’s modified Eagle’s medium (DMEM 4.5 g/L). The normal BJ cell line was also purchased from ATCC (Manassas, VA, USA) and was cultivated in low-glucose Dulbecco’s modified Eagle’s medium (DMEM 1 g/L). The media was supplemented in both cases with 10% FBS. Cells were cultured in T75 flasks and incubated at 37 °C in a humidified incubator with 5% CO_2_ supplementation. For maintenance, the media was changed every 2–3 days, and when the confluence of the flask reached approximately 85%, the cells were either subcultured or further used in the experiments.

#### 2.4.2. Cell Viability

ARPE-19 cells were seeded at a density of 2 × 10^4^ cells/well and 1 × 10^4^ cells/well (suspended in 100 µL) in 96-well plates for 24 h and 48 h, respectively. For the BJ cells, the seeding density was 0.5 × 10^4^ cells/well and 0.3 × 10^4^ cells/well for 24 h and 48 h, respectively, and for the A549 cells, 1.5 × 10^4^ cells/well for 24 h and 0.75 × 10^4^ cells/well for 48 h. As cells have different sizes and volumes, the different seeding densities were selected to reach a confluency of 80–90% by the end of the experiments. Cells were further exposed to the synthesized compounds for 24/48 h at concentrations ranging from 12.5 to 500 µM. After the incubation period, the exposure media was removed, the cells were washed with PBS, and further incubated with a 200 µM resazurin solution for 3 h as described in our previous report [108]. The conversion of resazurin to the fluorescent resorufin compound by viable cells was measured at λ_excitation_ = 550; λ_emission_ = 600, using SpectraMax iD3 (Molecular Devices, San Jose, CA, USA). The experiments were conducted in biological triplicate, each with a minimum of 3 technical replicates. The measured viability was expressed as relative values to the control (cells exposed only to the vehicle–culture medium containing 0.4% DMSO).

#### 2.4.3. Statistical Analysis

Statistical analyses were performed using SigmaPlot 15.0 software (Systat, Software Inc., Chicago, IL, USA). Results were analyzed for statistical significance using one-way analysis of variance (ANOVA) followed by a post hoc Holm–Sidak test. The significance level was set at *p* < 0.05. The data were represented as the mean ± SD (standard deviation) from at least three independent experiments.

## 3. Results

### 3.1. Chemical Synthesis

Following the outlined steps in the synthesis protocol for the isolation and purification of intermediate compounds **3a**,**b** and the final compounds **5a**,**b**, spectral data were recorded to verify the successful obtainment of the compounds. In each mass spectrum recorded for the intermediate compounds **3a**,**b** and the final compounds **5a**,**b**, the signals corresponding to the molecular peak were identified. In the ^1^H-NMR spectra recorded for the intermediate compounds **3a**,**b** and the final compounds **5a**,**b**, all peaks, with expected multiplicity and integrals, were identified and assigned to the protons from the molecules. No fractional integrals were found in the ^1^H-NMR spectra. The intermediate compounds **3a**,**b** were previously reported in the literature by other research groups, and the analysis performed by our group is consistent with the respective data [75,109,110,111]. All spectra data recorded are presented in the Supplementary Materials.

*(E)-2-((E)-but-2-en-1-ylidene)hydrazine-1-carbothioamide* (**3a**): pale yellow solid; yield = 49%; FT IR (KBr) ν_max_cm^−1^: 3384 (C-H), 3167 (C-H), 1650 (C=C), 1603 (C=N); MS: *m/z* = 144.1 (M + 1); ^1^H-NMR (DMSO-*d*_6_, 500 MHz) δ: 11.144 (s, 1H, N-H), 8.043 (br, 1H, H-N-H), 7.697 (d, 1H, -CH=N, *J* = 8.50 Hz), 7.500 (br, 1H, H-N-H), 6.197–6.083 (m, 2H, -CH=CH-), 1.836 (d, 1H, -CH_3_, *J* = 5.50 Hz); ^13^C-NMR (DMSO-*d*_6_, 125 MHz) δ: 177.599 (C=S), 144.856 (C=N), 138.172 (-CH=CH-), 128.401 (-CH=CH-), 18.393 (-CH_3_).

*(E)-2-((E)-4-(2,6,6-trimethylcyclohex-1-en-1-yl)but-3-en-2-ylidene)hydrazine-1-carbothioamide* (**3b**): yellow solid; yield = 73%; FT IR (KBr) ν_max_cm^−1^: 3397 (C-H), 3261 (C-H), 2947 (C-H), 2924 (C-H), 2854 (C-H), 2815 (C-H), 1647 (C=C), 1606 (str C=N), 1507 (C=C); MS: *m/z* = 266.2 (M + 1); ^1^H-NMR (DMSO-*d*_6_, 500 MHz) δ: 10.138 (s, 1H, N-H), 8.163 (br, 1H, H-N-H), 7.747 (br, 1H, H-N-H), 6.586 (d, 1H, -CH=, *J* = 17.00 Hz), 6.123 (d, 1H, -CH=, *J* = 16.50 Hz), 2.062 (s, 3H, -CH_3_), 2.018 (t, 2H, *J* = 6 Hz), 1.691 (s, 3H, ionone-CH_3_), 1.596–1.572 (m, 2H, -CH_2_-), 1.461–1.438 (m, 2H, -CH_2_-), 1.028 (s, 6H, CH_3_ and -CH_3_); ^13^C-NMR (DMSO-*d*_6_, 125 MHz) δ: 178.537 (C=S), 149.091 (C=N), 136.632 (cyclohexene C=), 133.027 (cyclohexene C=), 132.348 (cyclohexene C=), 130.431(-CH=), 39.174 (cyclohexene -CH_2_-), 33.757 (cyclohexene -CH_2_-), 32.532 (cyclohexene -C(CH_3_)(CH_3_)-), 28.668 (ionone-CH_3_), 21.403 (ionone-CH_3_), 18.610 (cyclohexene -CH_2_-), 11.821 (-CH_3_-C=N).

The peaks that originate from the aliphatic fragment of the thiosemicarbazones **3a**,**b** were easily identifiable in both ^1^H-NMR and ^13^C-NMR spectra of the final thiazoles **5a**,**b**. Successful cyclization of the thiosemicarbazones **3a**,**b** to the final thiazole compounds **5a**,**b** was confirmed by the disappearance of the three N-H peaks (most of them being broad signals) and the appearance of the characteristic singlet corresponding to the proton from position 5 of the newly obtained thiazole ring at approximately 6.9 ppm and of the three aromatic protons from the newly inserted catechol with characteristic splitting—one of them as a doublet of doublets and two as doublets. In the ^13^C-NMR spectra recorded for the final compounds **5a**,**b**, all signals were identified in the expected region and were assigned to the carbon atoms of the molecules. The peak of the strongly deshielded C=S at approximately 177 ppm from the **3a**,**b** thiosemicarbazones disappeared in the final **5a**,**b** thiazoles, consequently identifying in the ^13^C-NMR spectra of the final compounds **5a**,**b** the characteristic signals of the thiazole from position 5 at approximately 100 ppm and the new aromatic carbon atoms from the catechol.

*4-(2-(2-((1E,2E)-but-2-en-1-ylidene)hydrazineyl)thiazol-4-yl)benzene-1,2-diol hydrochloride* (**5a**): yellow solid; decomposition over 250 °C; yield = 61%; FT IR (KBr) ν_max_cm^−1^: 3443 (C-H), 3320 (C-H), 3091 (N-H), 2418 (C-H), 2365 (C-H), 2357 (C-H), 2348 (C-H), 2327 (C-H), 2310 (C-H), 1653 (C=C), 1624 (C=N), 1521 (C=N), 1294 (OH), 1239 (OH); MS: *m/z* = 274.0 (M − 1); ^1^H-NMR (DMSO-*d*_6_, 500 MHz) δ: 7.896–7.879 (m, 1H, -CH=N), 7.198 (d, 1H, Ar, *J* = 2.00 Hz), 7.087 (dd, 1H, Ar, *J* = 8.00 Hz and *J* = 2.00 Hz), 6.970 (s, 1H, ThC5), 6.798 (d, 1H, Ar, *J* = 8.00 Hz), 6.216–6.202 (m, 2H, -CH=CH-), 1.851–1.847 (m, 3H, -CH_3_); ^13^C-NMR (DMSO-*d*_6_, 125 MHz) δ: 168.066 (-CH=N), 147.418 (ThC4), 146.039 (Ar-OH), 145.332 (Ar-OH), 138.116 (-CH=), 128.121 (=CH-), 123.774 (Ar), 117.426 (Ar), 115.774 (Ar), 113.618 (Ar), 100.803 (ThC5), 18.393 (-CH_3_).

*4-(2-(2-((2E,3E)-4-(2,6,6-trimethylcyclohex-1-en-1-yl)but-3-en-2-ylidene) hydrazineyl) thiazol-4-yl)benzene-1,2-diol hydrochloride* (**5b**): red solid; decomposition over 180 °C; yield = 63%; FT IR (KBr) ν_max_cm^−1^: 3446 (C-H), 3351 (C-H), 3121 (N-H), 2955 (C-H), 2926 (C-H), 2872 (C-H), 2815 (C-H), 2418 (C-H), 2373 (C-H), 2365 (C-H), 2351 (C-H), 2344 (C-H), 2332 (C-H), 1647 (C=C), 1619 (C=N), 1521 (C=N), 1507 (C=C), 1362 (OH), 1267 (OH); MS: *m/z* = 396.0 (M − 1); ^1^H-NMR (DMSO-*d*_6_, 500 MHz) δ: 7.220 (d, 1H, Ar, *J* = 2.00 Hz), 7.114 (dd, 1H, Ar, *J* = 8.00 Hz and *J* = 2.00 Hz), 6.961 (s, 1H, ThC5), 6.773 (d, 1H, Ar, *J* = 8.00 Hz), 6.561 (d, 1H, ionone-CH=, *J* = 16.50 Hz), 6.091 (d, 1H, =CH-, *J* = 16.50 Hz), 2.123 (s, 3H, -CH_3_-C=N), 2.040–2.015 (m, 2H, -CH_2_-), 1.714 (s, 3H, ionone-CH_3_), 1.619-1.570 (m, 2H, -CH_2_-), 1.473-1.449 (m, 2H, -CH_2_-), 1.042-1.039 (m, 6H, -CH_3_ and -CH_3_); ^13^C-NMR (DMSO-*d*_6_, 125 MHz) δ: 168.787 (ThC2), 145.591 (Ar-OH), 145.248 (Ar-OH), 136.660 (cyclohexene C=), 132.537 (cyclohexene C=), 130.263 (-CH=), 117.083 (Ar), 115.662 (Ar), 113.317 (Ar), 100.887 (ThC5), 39.006 (cyclohexene -CH_2_-), 33.813 (cyclohexene -CH_2_-), 32.504 (cyclohexene -C(CH_3_)(CH_3_)-), 28.724 (ionone-CH_3_), 21.459 (ionone-CH_3_), 18.645 (cyclohexene -CH_2_-), 12.262 (-CH_3_-C=N).

### 3.2. In Vitro Antioxidant Evaluation

#### 3.2.1. Radical Scavenging Assays

The power of compounds **5a**,**b** to scavenge radicals 1,1-diphenyl-2-picrylhydrazyl (DPPH^•^) and 2,2′-azino-bis(3-ethylbenzothiazoline-6-sulfonic acid) (ABTS^•+^), as seen in Table 1, is expressed as half-maximal inhibitory concentrations (IC_50_), along with the reference compound, Trolox. In both DPPH^•^ and ABTS^•+^ radical scavenging assays, both compounds **5a**,**b** exhibited high antiradical activity when compared to the reference compounds ascorbic acid and Trolox (up to 5-fold higher activity of compound **5a** in the DPPH^•^ scavenging assay). Compound **5a** was slightly more active than **5b**, a consequence of the different aliphatic substituent from the azomethine, since this is the only difference between the two compounds. Compound **5a** is an aldehyde hydrazone, while compound **5b** is a ketone hydrazone, a molecular design that explains well the difference in reactivity between the two compounds and which was previously identified by our group in compounds with a similar structure [112]. It seems that the smaller but-2-enylidene aldehyde hydrazone (**5a**) is preferred to the larger β-ionone hydrazone (**5b**) when comparing the antiradical DPPH^•^ and ABTS^•+^ activity of the compounds.

**Figure 9:**
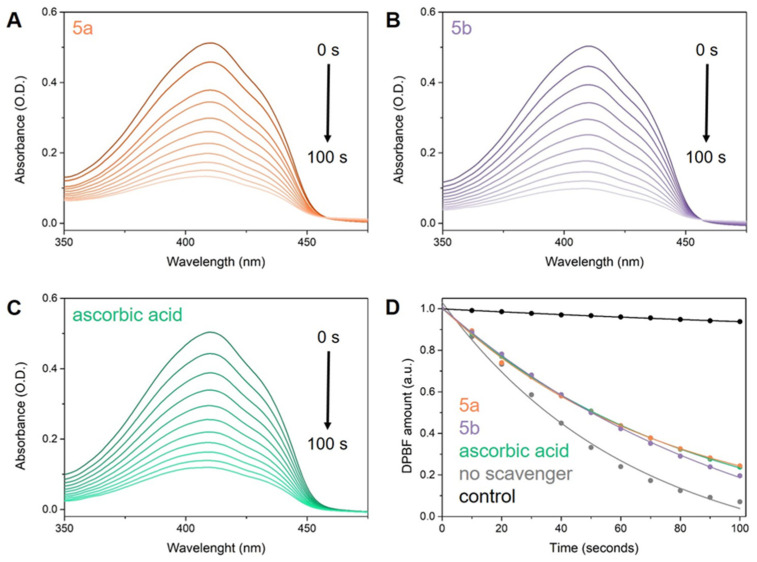
Degradation of DPBF over a 100-s irradiation period, as monitored by UV-Vis absorption spectra in the presence of ICG and (**A**) **5a**, (**B**) **5b**, and (**C**) ascorbic acid. Additionally, (**D**) presents fitted plots depicting the DPBF amount after irradiation in the presence of ICG and tested ^1^O_2_ scavengers: **5a** (orange), **5b** (purple), ascorbic acid (green), and without any scavenger (grey).

The efficacy of compounds **5a**,**b** against ^1^O_2_ was assessed using an indirect approach. ICG was used as a photo-induced ^1^O_2_ generator alongside DPBF, serving as an indirect chemical probe capable of characterizing ^1^O_2_ release in solution. This setup allowed us to evaluate the ^1^O_2_ scavenging capacity of the tested molecules and compare their activity to a reference compound, specifically ascorbic acid. The samples were exposed to 785 nm laser radiation, and at 10-s intervals after irradiation, their UV-Vis absorption spectra were measured and analyzed (Figure 9). Subsequently, we determined the ^1^O_2_ quantum yields (Φ(^1^O_2_)) of ICG in the presence of **5a**,**b** and ascorbic acid, using the Φ(^1^O_2_) of free ICG of 15.0% as reference.

Our study confirms the outstanding ^1^O_2_ scavenging capacity of the compounds **5a**,**b**, as evidenced by the inverse relationship between antioxidant activity and the quantum yield of ^1^O_2_ production in molecules. As anticipated, the addition of ascorbic acid to ICG (Φ(^1^O_2_) of 9.4%) reduces its ^1^O_2_ quantum yield by 5.6% compared to ICG alone (Φ(^1^O_2_) of 15% [91]), validating our approach. Moreover, the tested molecules exhibit comparable ^1^O_2_ scavenging activity to ascorbic acid, with **5a** demonstrating a Φ(^1^O_2_) of 11.1% and **5b** exhibiting a Φ(^1^O_2_) of 11%.

#### 3.2.2. Electron Transfer Assays

Through the methods of TAC, RP, and FRAP spectrophotometric assays, the antioxidant capacity of **5a**,**b** was determined based on their ease to donate electrons. Both compounds exhibited higher activity than the reference compounds in all assays, with compound **5a** exhibiting higher activity than **5b** (Table 2). The overall identified activity for the compounds is different and is mainly influenced by the oxidation environment of each assay—different pH, different oxidizing agent, or different temperature. This is why performing more electron transfer assays helps in obtaining a clearer and more detailed assessment of the antioxidant properties of the tested compounds, leading to more reliable and comprehensive conclusions.

#### 3.2.3. Ferrous Ions Chelation Assay

The capacity of compounds **5a**,**b** to chelate the ferrous ions was evaluated and compared with EDTA-Na_2_ in an assay using ferrozine as a chromogen chelator of the free ferrous ions. The results of the ferrous ions chelation assay are presented in Table 3.

#### 3.2.4. Lipid Peroxidation Inhibition Assay (LPI)

Lipid peroxidation inhibition was analyzed using the rat microsomal membrane assay employing the 2-thiobarbituric acid method for measuring the amount of malondialdehyde resulting from the reaction and was expressed as IC_50_. Table 4 presents the results for compounds **5a**,**b**, in comparison with the reference agent Trolox. Both compounds displayed a superior capacity for inhibition, with compound **5a** being approximately 7.5-fold more active than Trolox.

### 3.3. In Silico Studies

#### 3.3.1. Quantum and Thermodynamics Calculations

Frontier molecular orbitals are fundamental descriptors in chemistry, specifically addressing electron donation (HOMO) and electron acceptance (LUMO). In the context of antioxidant activity, the HOMO-LUMO levels and the energy gap between them serve as indicators of a compound’s electron-donating or accepting capabilities, essential for its role as an antioxidant. The representation of these two orbitals and their respective energies for compounds **5a**,**b** is presented in Table 5.

The distribution of the frontier orbitals is similar between the two compounds. HOMO is distributed over the aliphatic double bond(s)—hydrazone-thiazole-catechol fragment (not on the aliphatic unconjugated area of the ionone), while LUMO is found over the aliphatic double bond(s)—hydrazone-thiazole fragment (not on the catechol and the aliphatic unconjugated area of the ionone).

Both frontier orbitals of compound **5b** have slightly lower energy than **5a** in all solvents, which makes it a better nucleophile and electrophile. Still, the differences in the energy levels of the frontier molecular orbitals of the two compounds are small. The difference in reactivity between **5a** and **5b** is difficult to attribute only to the HOMO and LUMO orbital energies (Table 6).

Considering the potential antiradical mechanism of the compound by breaking the heteroatom-H bond to release hydrogen atoms, the ease of breaking the respective bonds from three potential sites was calculated: N-H from hydrazone and both O-H from catechol (Table 6). The most suitable group to release the hydrogen atom is the OH phenolic group from the *para* position relative to the linker, compound **5b** having lower values than compound **5a**.

#### 3.3.2. ADME Study

The obtained data predicts the molecular properties of compounds **5a**,**b** by describing their physicochemical characteristics, lipophilicity, solubility, pharmacokinetics, and druglikeness using the SwissADME platform. The results are presented in Table 7 and Table 8.

According to Lipinski’s rule of five, the molecular weights of compounds **5a**,**b** were 275.33 or 397.74 g/mol (<500 g/mol), with 4 or 5 rotatable bonds (<10), a number of 4 HBA bonds (<10) and 3 HBD bonds (<5), TPSA value of 105.98 Å^2^ (<140), and logP values of 3.23 or 4.77 (<5) [113]. Also, the logS value was predicted to be −3.28 or −6.00 and evaluated by the following: −10 (insoluble), −6 (poorly soluble), −4 (soluble), −2 (extremely soluble), and 0 (highly soluble) [107].

In line with the anticipated pharmacokinetic properties, compound **5a** is potentially highly absorbed via oral administration, while compound **5b** is likely to have low GI absorption. Both compounds, **5a** and **5b**, do not cross the blood-brain barrier and are not substrates for P-glycoprotein. Investigating 5 isoforms of CYP450, it was found that compound **5a** inhibits none of these, while compound **5b** inhibits CYP2C9, CYP2C19, and CYP3A4 isoforms [107].

### 3.4. In Vitro Cytotoxicity Evaluation

**Figure 10:**
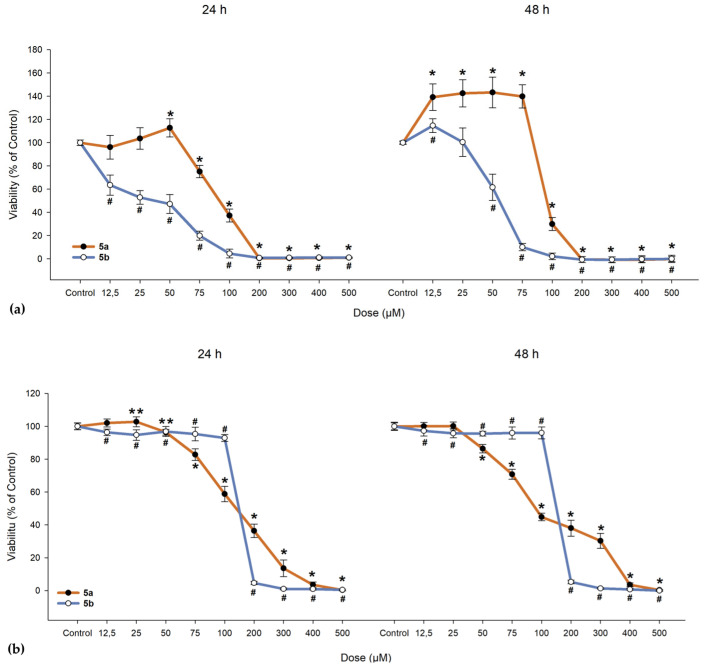

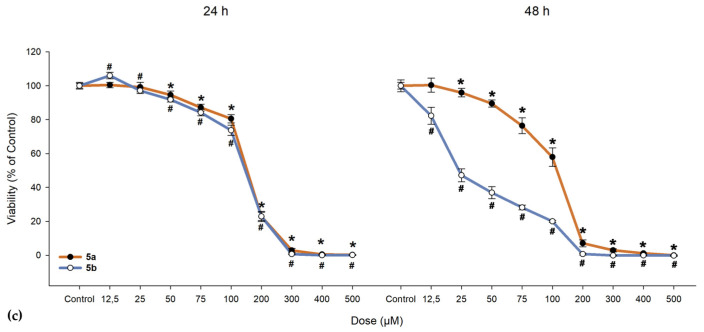
Cell viability of (**a**) human retinal pigment epithelial cells (ARPE-19), (**b**) dermal fibroblasts (BJ), and (**c**) lung adenocarcinoma cells (A549). Cells were exposed to different concentrations of **5a** and **5b**. Data (% of the control group) are expressed as mean ± SD of three independent experiments with a minimum of three technical replicates/experiment. ** *p* < 0.02, * *p* < 0.001 vs. control (for **5a**); # *p* < 0.001 vs. control (for **5b**).

We investigated the cytotoxic effects of **5a** and **5b** on ARPE-19 cells alongside a cancerous line (A549) and normal fibroblast cells (BJ). As shown in Figure 10, **5a** exhibited a slight proliferative effect on ARPE-19 cells up to 50 µM, beyond which the compound became toxic, leading to cell death. When exposed to **5b**, ARPE-19 cells began to die at much lower doses (as evidenced by the 24-h exposure, where the lowest dose of 12.5 µM resulted in a drop to 63.46% in cell viability; IC_50_ = 31.47 µM, as seen from Table 9). One-way ANOVA revealed significant differences between nearly all concentrations of **5a** and **5b** when compared to the control group (Figure 10). To determine whether the compounds maintain the same proliferative profile, BJ and A549 cells were also treated with these compounds. In both the dermal fibroblasts and cancerous cells, **5a** and **5b** exhibited similar toxic cutoffs, with a decrease of more than 50% in cell viability observed after the fifth dose in both exposure periods, showing no significant proliferative effect. Overall, cells responded better to **5a** compared to **5b**, with the ARPE-19 cell line demonstrating an increase in cell viability up to the third dose.

In the 48-h exposure setting, ARPE-19 displayed an increase in viability compared to the control, an effect that was absent in the BJ and A549 cell lines. All cell lines demonstrated near-null viability after exposure to doses exceeding 200 µM, regardless of the compound and exposure conditions.

## 4. Discussion

### 4.1. Chemical Synthesis

In the first step of the chemical synthesis, thiosemicarbazone intermediates **3a**,**b** were synthesized through a condensation reaction between compounds **1a** or **1b** and thiosemicarbazide (**2**) by refluxing in ethanol and using sulfuric acid as a catalyst. In the second step of the chemical synthesis, the final compounds **5a**,**b** were synthesized by a Hantzsch heterocyclization reaction between the intermediate thiosemicarbazones **3a**,**b** and 4-(chloroacetyl)catechol (**4**), by refluxing in acetone. Spectral analysis revealed the successful obtainment of the intended compounds.

### 4.2. In Vitro Antioxidant Evaluation

The results of all the performed in vitro assays showed that compounds **5a**,**b** exhibited a higher antioxidant activity than both reference compounds (ascorbic acid and Trolox). A negligible chelation capacity for the ferrous ions was identified, lower than that of the EDTA used as a reference. Thus, the present compounds possess antioxidant activity but lack the complementary mechanism to chelate ferrous ions and are unable to prevent undesired Fenton-type reactions, catalyzed by the respective free ions.

In all the other antioxidant assays, the most active compound was **5a**, the 2-butenal derivative, while compound **5b**, the β-ionone derivative, exhibited lower antioxidant activity. Also, compound **5a** expressed a stronger inhibition of lipid peroxidation than compound **5b**. This can be attributed to the relatively large differences in terms of size and electronic effects of the substituents grafted on the azomethine group when comparing the two compounds.

### 4.3. In Silico Studies

#### 4.3.1. Quantum and Thermodynamics Calculations

Density Functional Theory (DFT) has revolutionized the field of antioxidants` design, providing a robust theoretical framework for understanding molecules at the atomic level through electronic property calculations. This makes it essential for predicting their antioxidant behavior. By conducting simulations that scrutinize the electronic structure and energy of molecules, as well as the potential radicals that may arise, DFT emerges as a crucial tool for identifying potent antioxidants with effective free radical scavenging capabilities.

The decision to conduct evaluations in multiple environments was motivated by the potential to get valuable insights into the behavior and efficacy of the compounds under varied conditions. The interaction of the studied molecules with solvent molecules possessing distinct chemical and electronic properties holds substantial influence over the compound’s behavior.

Regarding the HOMO-LUMO frontier orbitals, compound **5b** showed slightly lower energy than **5a** in all solvents (gas, nonpolar solvent (ε = 7.43), and water), describing better nucleophile and electrophile properties than compound **5a**. Because the energy levels of these frontier orbitals were not enough to analyze the reactivity difference between compounds **5a**,**b**, the BDE values were also calculated.

Overall, the group that would release the hydrogen atom the easiest is the phenol group from *para*, independent of the compound and environment, with a BDE between 66.35 kcal/mol and 69.74 kcal/mol. Depending on the compound and environment, the heterolytic breaking of the N-H bond and the *meta* O-H would occur between 68.41 kcal/mol and 73.87 kcal/mol.

The solvent negatively affects the heterolytic breaking of the bonds in compounds **5a**,**b**, increasing the BDE between 1.24 kcal/mol and 2.70 kcal/mol. Between the two compounds, the difference of BDE for a specific phenol group in all solvents is negligible, but a significant difference is found for the BDE of the N-H bond in all solvents, being higher for **5a**, and ranging between 1.79 kcal/mol and 2.01 kcal/mol.

#### 4.3.2. ADME Study

The synthesized compounds were predicted to have suitable druggability properties, as Lipinski’s rule of five was not violated. Regarding bonds, the only difference observed was in the number of rotatable bonds, which is equal to 5 for the β-ionone-derived compound **5b** and 4 for the 2-butenal-derived compound **5a**. The TPSA descriptor had the same value, meaning that the polarity of these compounds was not influenced by the substituent’s nature. Analyzing TPSA and logP values, these compounds have a moderate lipophilic-hydrophilic profile, which can moderately overpass lipid membranes. According to the literature, at the obtained logP values, the compounds should cross the blood-retina barrier. It is not essential for them to also pass the blood-brain barrier. Additionally, logP values less than 5 and molecular weight values less than 500 g/mol were found to be suitable for this purpose [114,115]. LogS values indicate low solubility, but the obtained results are favorable for compound **5a**.

The pharmacokinetic properties of the designed compounds were described using specific descriptors: GI absorption, blood-brain barrier permeability, P-gp substrates, and inhibition of the most important CYP450 isoenzymes. In terms of absorption, compound **5a** is suitable for oral administration as confirmed by the GI absorption values and the better solubility properties. Neither compound was predicted to cross the BBB, limiting the risk of unwanted brain-related side effects after systemic administration. Neither compound was predicted to be a substrate for P-gp, which could suggest a low elimination rate. The elimination phase of these compounds, which is not directly under P-gp efflux, is probably influenced indirectly by the interaction with other transporter proteins. Only compound **5b** is expected to alter the metabolism of other drugs by inhibiting CYP2C19, CYP2C9, and CYP3A4 [107,114,116].

### 4.4. In Vitro Cytotoxicity Evaluation

After confirming the compounds have potent antioxidant activity, we next focused our attention on assessing potential toxicity concerns in RPE cells, as well as normal skin fibroblasts and lung cancer cells. Host tissue toxicity assessment of new compounds is an important step early in the drug discovery process and was performed by evaluating cell viability using the resazurin salt solution. The ARPE-19 cell line has been selected as these cells are a good cellular model for studying RPE physiology and degenerative ocular pathologies involving the retina because of their characteristics akin to native RPE cells, while BJ and A549 cells were selected to study possible systemic implications and because β-carotene was found to increase the risk of lung cancer in smokers, which eventually led to it being replaced with lutein and zeaxanthin in the Age-Related Eye Disease Study 2 (AREDS 2) supplement formulation [117].

Cell viability of ARPE-19 cells was more easily influenced when compared to BJ and A549 cells at the same concentration of either **5a** or **5b**. An unexpected effect was identified in both exposure settings where **5a** at lower doses has a proliferative effect when compared to any dose of **5b**, suggesting a potential protective role that could contribute to repairing RPE layer defects—an essential aspect of AMD pathology. A549 cells were not seen to proliferate, suggesting a possible safer profile than β-carotene regarding lung cancer. As seen from Figure 10, doses that induced BJ cell death were much higher than the ones for the ARPE-19 cells, suggesting that a dose biocompatible with the retina cells would have low systemic side effects.

Another surprising effect was the high toxicity of **5b**, as this compound was created using the β-ionone moiety, known for its beneficial effects on the RPE layer mediated through the OR [63,65]. The human RPE performs a variety of functions essential for visual perception, from light absorption to phagocytosis and transepithelial transport. Most of these functions are regulated via G protein-coupled receptors (GPCRs) [118]. The genes coding for the OR, which also represent the largest GPCR family in the human genome [119], were first described in the olfactory epithelium of the rat, where, upon stimulation, they increased intracellular calcium levels. OR expression is found in various human tissues—colon, ovary, liver, kidney, lymph node, testis, prostate, melanocytes, and more recently RPE [65,120,121,122,123,124]. The physiological role of these ectopically expressed ORs is the subject of ongoing research. It has been shown that their stimulation in prostate cancer cells inhibits proliferation and induced invasiveness [65] and inhibits proliferation and migration of melanocytes as well as induces melanogenesis [65], thus affecting cell-type-specific physiological processes. Regarding RPE cells, Jovancevic et al. demonstrated that the β-ionone activation of OR51E2 resulted in increased proliferation and metastasis by activating p44/42 and protein kinase B (AKT) proteins. The group also found that the threshold concentration to trigger a cellular response by β-ionone was under 10 µM [124], while in our study, high cellular toxicity for **5b** was observed already at 12.5 µM.

While protective effects against induced retinal toxicity were not assessed, such investigations are planned in future work. These upcoming studies will utilize the data presented here to evaluate the compounds’ efficacy in biologically relevant disease models for AMD pathogenesis.

## 5. Conclusions

In this paper, two new unreported compounds were successfully synthesized using either 2-butenal or β-ionone as raw material and verified by a series of characterization data. In vitro antioxidant experiments showed excellent antioxidant activity of the final compounds **5a**,**b** in comparison with the reference antioxidant agents, ascorbic acid and Trolox. The hydrazone-catechol-thiazole scaffold gave a higher antioxidant potential, compound **5a**, with the 2-butenal moiety attached to the azomethine group, being the most active.

Frontier molecular orbitals, HOMO and LUMO, were distributed similarly between the two compounds, with HOMO being distributed over the aliphatic double bond(s)—hydrazone-thiazole-catechol fragment, while LUMO was found over the aliphatic double bond(s)—hydrazone-thiazole fragment. Also, the difference between **5a**,**b** in terms of the energy levels of these orbitals is low. The phenolic OH from the *para* position relative to the linker is expected to release hydrogen atoms the most efficiently, according to the BDE values, and exhibited the lowest values for compound **5b**. Regarding the predicted molecular properties, these compounds respect the Lipinski rules of five, having good druggability traits. Out of the two, only compound **5a** exhibited a suitable profile for oral administration as a potential pharmaceutical form.

The possible cytotoxicity at both a local (ARPE-19 cell line) and systemic (BJ and A549 cell lines) level was evaluated, and the results showed the promising proliferative effect of **5a** and the surprisingly highly toxic nature of **5b** despite its β-ionone moiety. To conclude, this study represents a foundational step within a broader research effort aimed at identifying protective agents against retinal degenerative processes. It lays the groundwork for upcoming studies by establishing the biological profile of the candidate compounds, notably **5a**, which displayed promising antioxidant activity and a potential protective effect due to its low-dose promotion of the proliferative response and its ability to mitigate oxidative stress, a significant mechanism in the pathogenesis of AMD.

## Figures and Tables

**Table 1:** The IC_50_ values identified for compounds **5a**,**b** against the DPPH^•^ and ABTS^•+^ scavenging assays.

Compound	DPPH^•^ IC_50_ (µM)	ABTS^•+^ IC_50_ (µM)
**5a**	9.80	4.67
**5b**	18.45	5.03
**Ascorbic acid**	50.21	N.T.
**Trolox**	35.77	15.87

N.T. = not tested.

**Table 2:** The results of the electron transfer assays conducted for compounds **5a**,**b**. Results are presented in terms of molar equivalents of reference compounds.

Compound	TAC	RP		FRAP
	Eq Ascorbic Acid	Eq Ascorbic Acid	Eq Trolox	Eq Trolox
**5a**	2.82	2.55	2.12	1.49
**5b**	2.56	1.90	1.58	1.39

**Table 3:** The results of the ferrous ions chelation assay (% of ferrous ions chelation).

Compound	50 nM	100 nM	150 nM	175 nM	200 nM
**5a**	−	−	−	−	−
**5b**	−	−	−	−	−
**EDTA-Na_2_**	11.71	20.00	36.37	61.98	86.53

“−“ represents less than 5% chelation activity.

**Table 4:** The IC_50_ values of the **5a**,**b** compounds and Trolox on inhibition of the rat microsomal membrane lipid peroxidation.

Compound	IC_50_ (µM)
**5a**	3.34
**5b**	12.90
**Trolox**	25.00

**Table 5:** Frontier molecular orbitals and electrostatic potential map (EPM) for compounds **5a**,**b**.

	5a	5b
**HOMO**	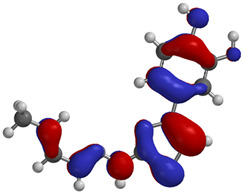	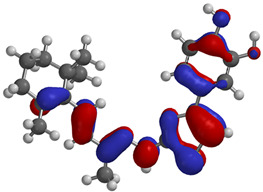
**LUMO**	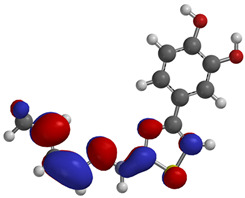	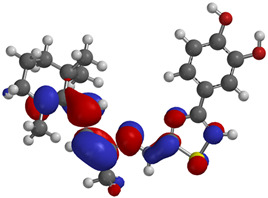
**EPM**	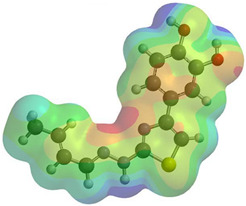	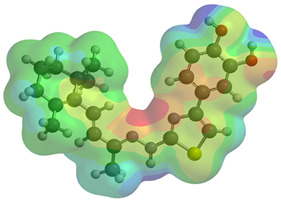

**Table 6:** Frontier molecular orbitals and N-H and O-H BDE of **5a**,**b** in gas, nonpolar (ε = 7.43) environment, and water.

Environment	Compound	Frontier Molecular Orbitals (eV)		Bond Dissociation Enthalpy (kcal/mol)		
		HOMO	LUMO	N-H	*p*O-H	*m*O-H
**gas**	**5a**	−4.96	−1.22	70.42	66.52	69.08
	**5b**	−4.89	−1.28	68.41	66.36	68.92
**nonpolar**	**5a**	−5.08	−1.30	72.46	68.40	71.53
	**5b**	−5.02	−1.35	70.67	68.48	71.62
**water**	**5a**	−5.11	−1.33	73.79	69.64	72.96
	**5b**	−5.07	−1.37	71.99	69.74	73.04

**Table 7:** Physicochemical properties, lipophilicity, solubility, and druglikeness of compounds **5a**,**b**.

Compounds	Physicochemical Properties					Lipophilicity	Solubility	Druglikeness
	Mw (g/mol)	Nrotbs	HBAs	HBDs	TPSA (Å^2^)	LogP	LogS	No. of Lipinski Violations
**5a**	275.33	4	4	3	105.98	3.23	−3.28	0
**5b**	397.53	5	4	3	105.98	4.77	−6.00	0

**Table 8:** Pharmacokinetic properties of compounds **5a**,**b**.

Compounds	Pharmacokinetics							
	GI abs	BBB	P-pg Substrate	CYP1A2 Inhibitor	CYP2C19 Inhibitor	CYP2C9 Inhibitor	CYP2D6 Inhibitor	CYP3A4 Inhibitor
**5a**	High	No	No	No	No	No	No	No
**5b**	Low	No	No	No	Yes	Yes	No	Yes

**Table 9:** The IC_50_ values of compounds **5a**,**b** regarding cytotoxicity.

Compound	Cell Line	24 h Exposure IC_50_ (µM)	48 h Exposure IC_50_ (µM)
**5a**	ARPE-19	90.1	92.7
	BJ	144.0	162.5
	A549	147.2	109.3
**5b**	ARPE-19	31.4	52.1
	BJ	141.4	152.0
	A549	166.8	39.6

## Data Availability

Data is contained within the article or Supplementary Materials.

## Supplementary Materials

The following supporting information can be downloaded at: https://www.mdpi.com/article/10.3390/antiox14060646/s1, Figure S1: The IR spectrum for the compound **3a**; Figure S2: The IR spectrum for the compound **3b**; Figure S3: The IR spectrum for the compound **5a**; Figure S4: The IR spectrum for the compound **5b**; Figure S5: The mass spectrum for the compound **3a**; Figure S6: The mass spectrum for the compound **3b**; Figure S7: The mass spectrum for the compound **5a**; Figure S8: The mass spectrum for the compound **5b**; Figure S9: The ^1^H-NMR spectrum for the compound **3a**; Figure S10: The ^1^H-NMR spectrum for the compound **3b**; Figure S11: The ^1^H-NMR spectrum for the compound **5a**; Figure S12: The ^1^H-NMR spectrum for the compound **5b**; Figure S13: The ^13^C-NMR spectrum for the compound **3a**; Figure S14: The ^13^C-NMR spectrum for the compound **3b**; Figure S15: The ^13^C-NMR spectrum for the compound **5a**; Figure S16: The ^13^C-NMR spectrum for the compound **5b**.
