# Supplementary material for: Antioxidant Activity and Cytotoxicity Evaluation of New Catechol Hydrazinyl-Thiazole Derivatives as Potential Protectors in Retinal Degenerative Processes
Source: Antioxidants (Basel). 2025 May 28;14(6):646. doi: 10.3390/antiox14060646 (PMC12189671; doi:10.3390/antiox14060646)
Supplement: Supplementary file 1 [file antioxidants-14-00646-s001.zip › antioxidants-3622226-supplementary.pdf]

antioxidants

MDPI

Supplementary Material: Antioxidant Activity and Cytotoxicity Evaluation of New Catechol Hydrazinyl-Thiazole Derivatives as potential protectors in retinal degenerative processes

Răzvan-Geo Antemie[1], Gabriel Marc[2*], Raluca Pele [2], Ionel Fizeșan[3], Ionuț-Valentin Creștin[3], Raluca Borlan[4], Panagiotis Theodosis-Nobelos[5], Eleni A. Rekka[6], Ovidiu Oniga[2], Ovidiu Crișan[7], Adrian Pîrnău[8], Laurian Vlase[9], and Simona Valeria Clichici[1]

1. Figures

1.1 The IR spectra

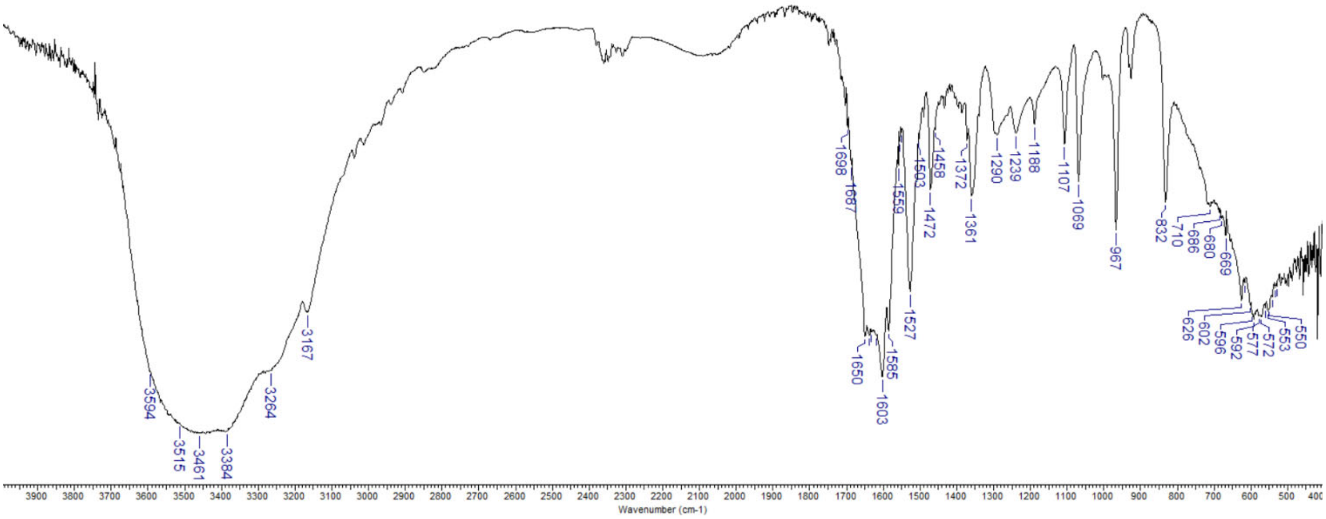

Figure S1. The IR spectrum for the compound **3a.**

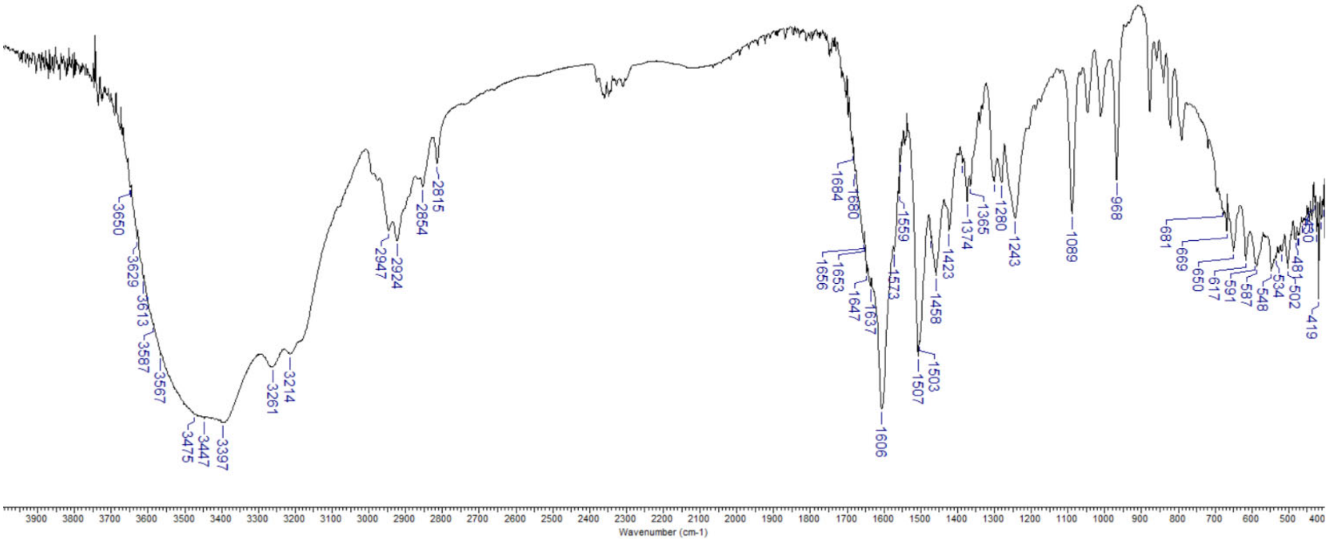

Figure S2. The IR spectrum for the compound **3b.**

 https://doi.org/10.3390/antiox14060646

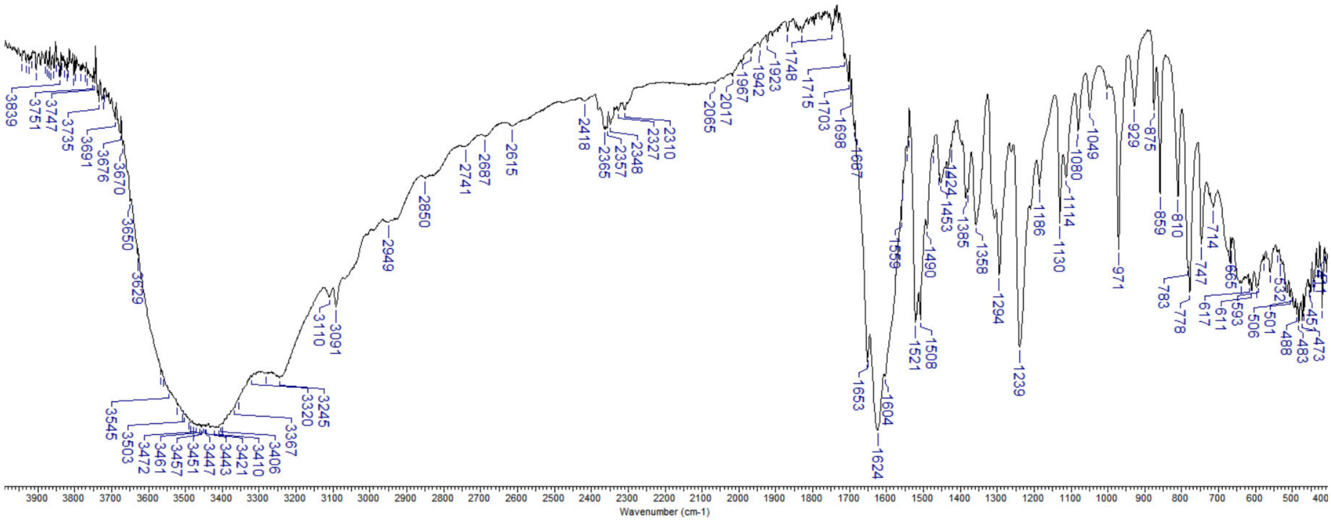

Figure S3. The IR spectrum for the compound **5a.**

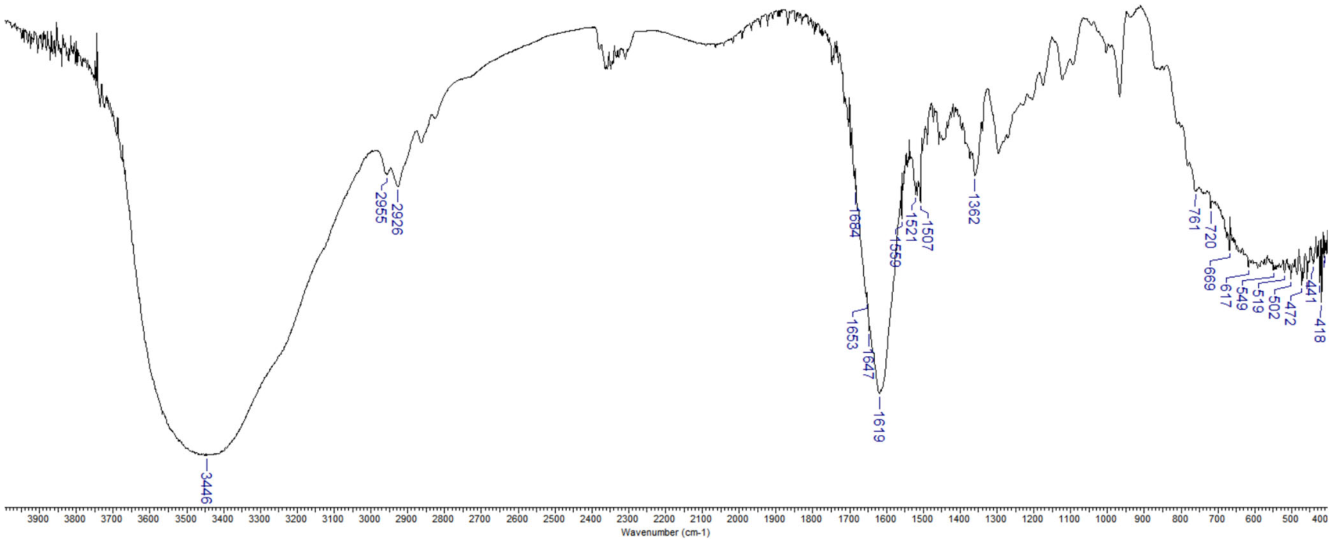

Figure S4. The IR spectrum for the compound **5b.**

1.2. The mass spectra

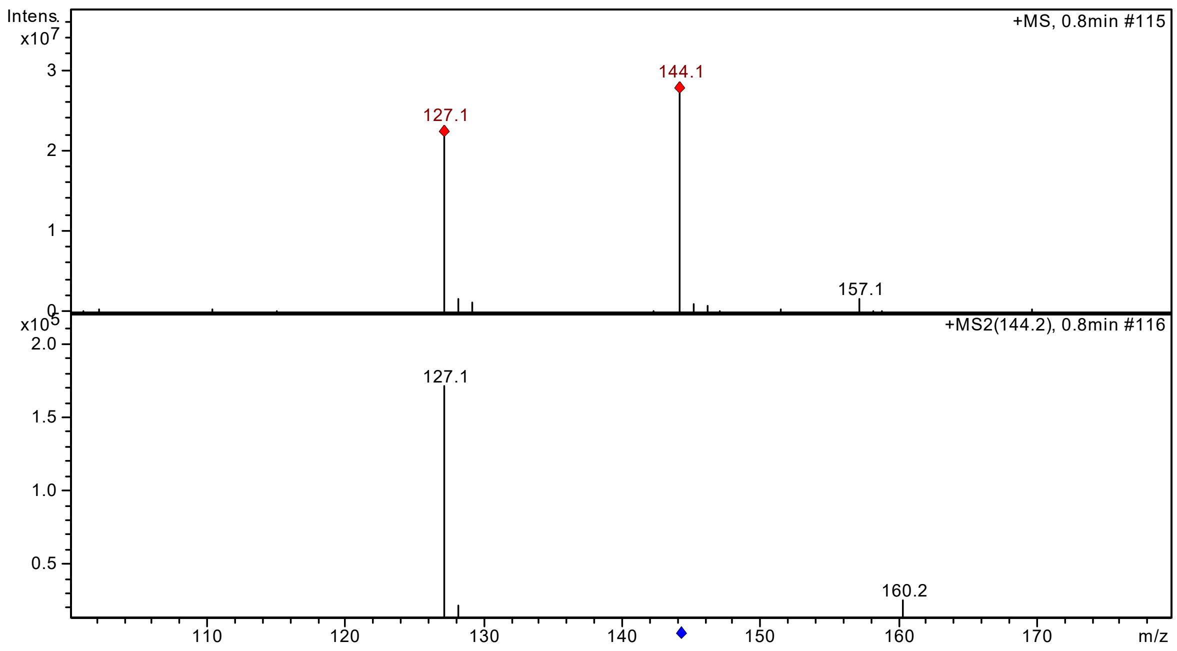

Figure S5. The mass spectrum for the compound **3a.**

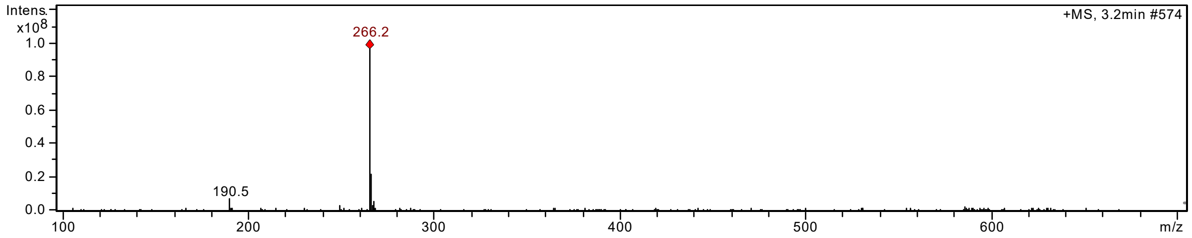

Figure S6. The mass spectrum for the compound **3b.**

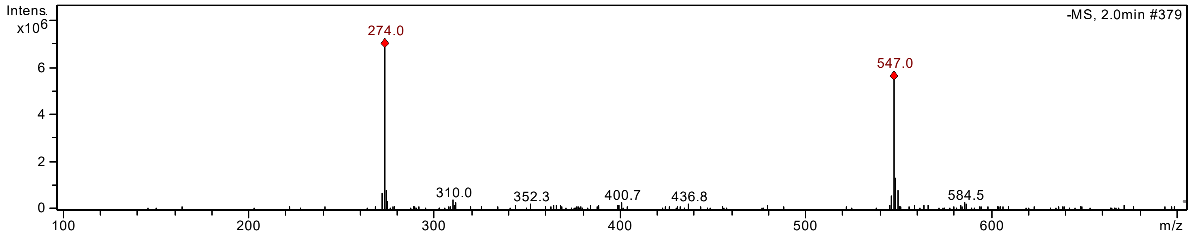

Figure S7. The mass spectrum for the compound **5a.**

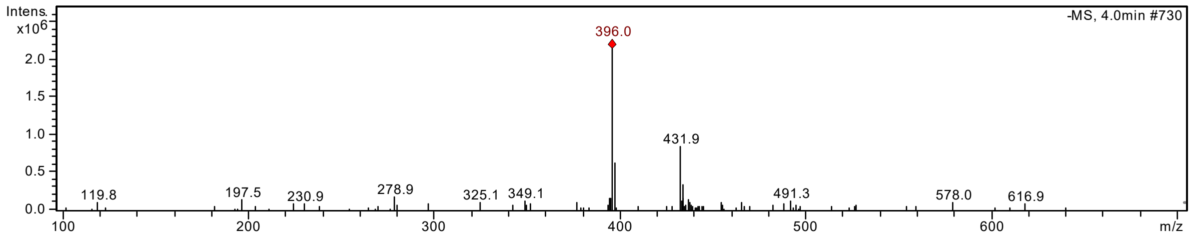

Figure S8. The mass spectrum for the compound **5b.**

1.3. The ^{1}H-NMR spectra

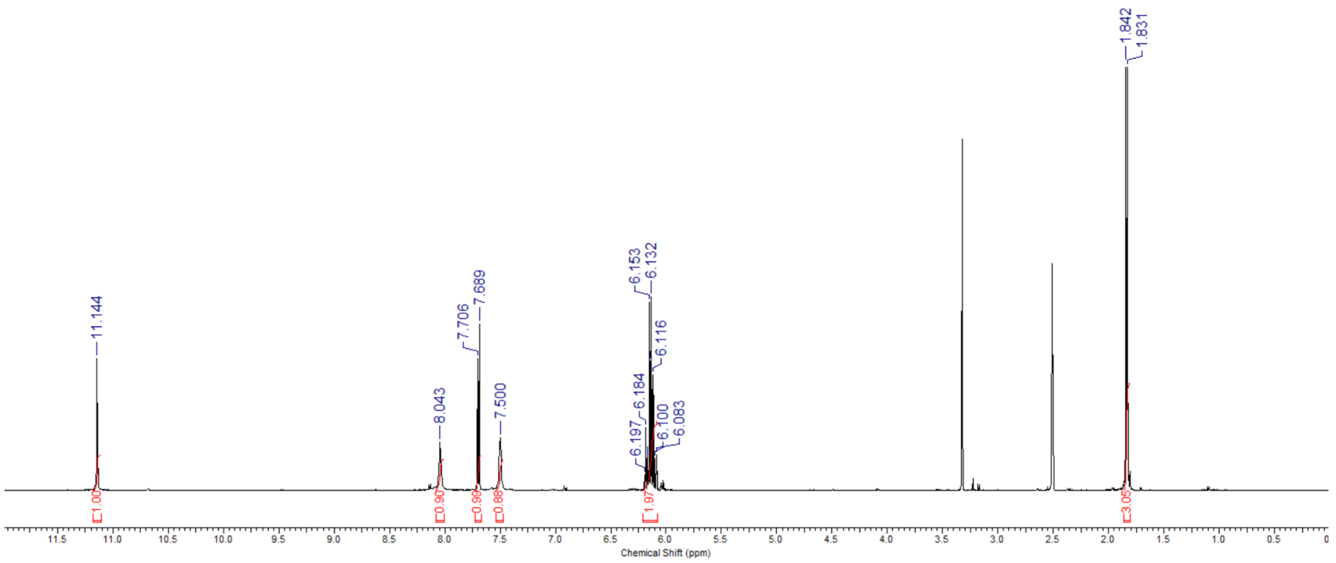

Figure S9. The ^{1}H-NMR spectrum for the compound **3a.**

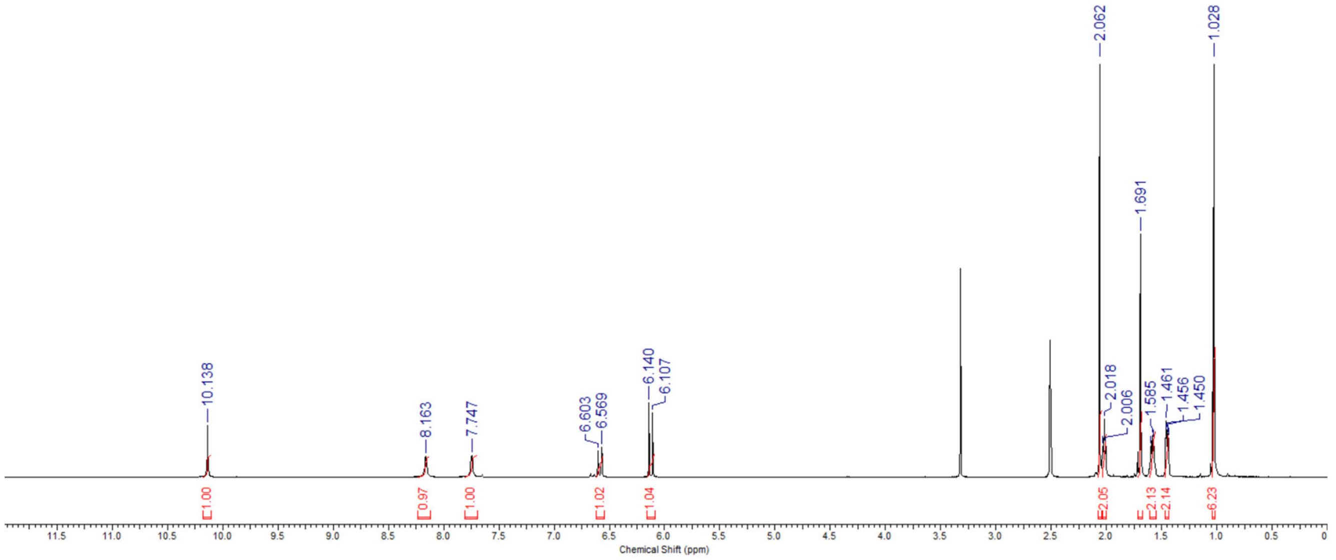

Figure S10. The ^{1}H-NMR spectrum for the compound **3b.**

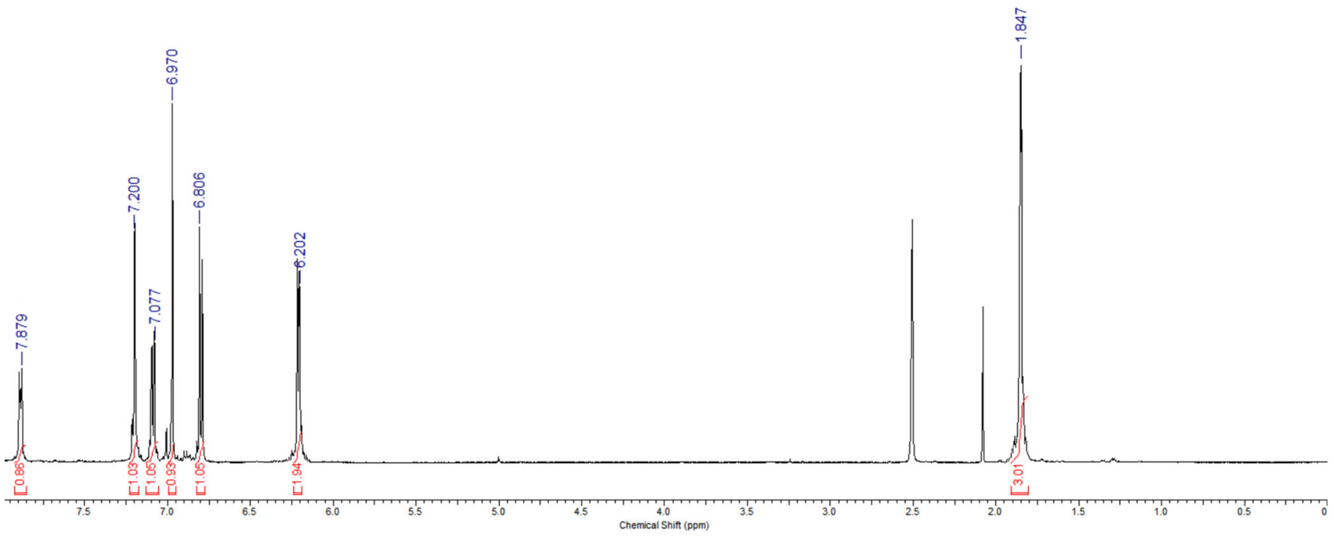

Figure S11. The ^{1}H-NMR spectrum for the compound **5a.**

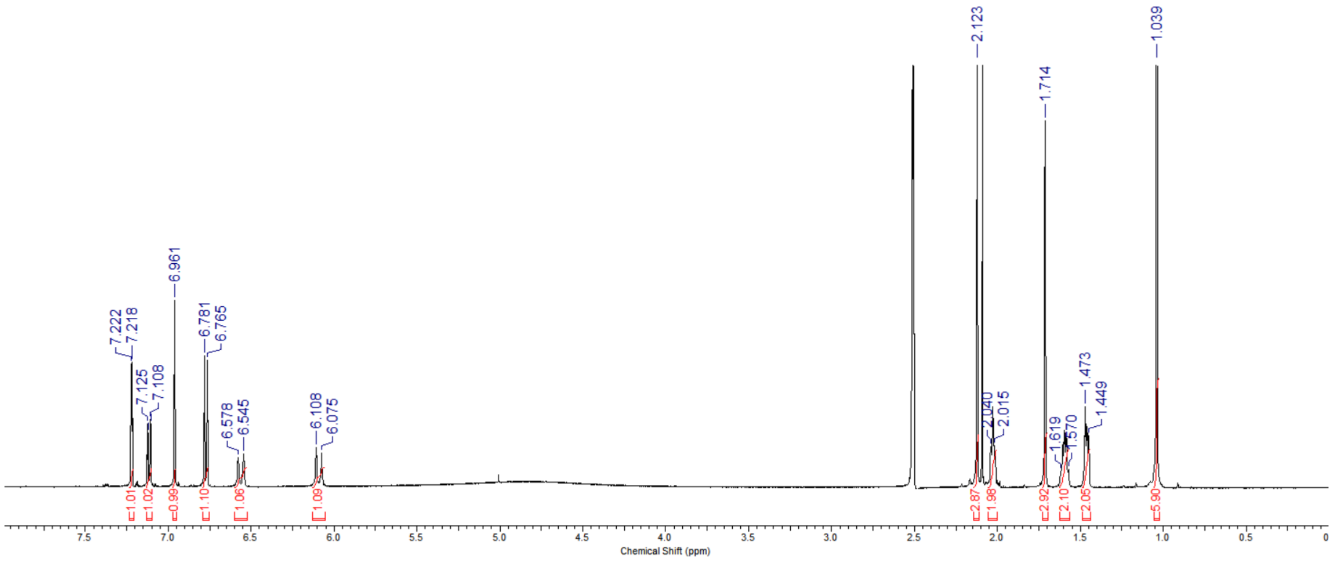

Figure S12. The ^{1}H-NMR spectrum for the compound **5b.**

1.4. The ^{13}C-NMR spectra

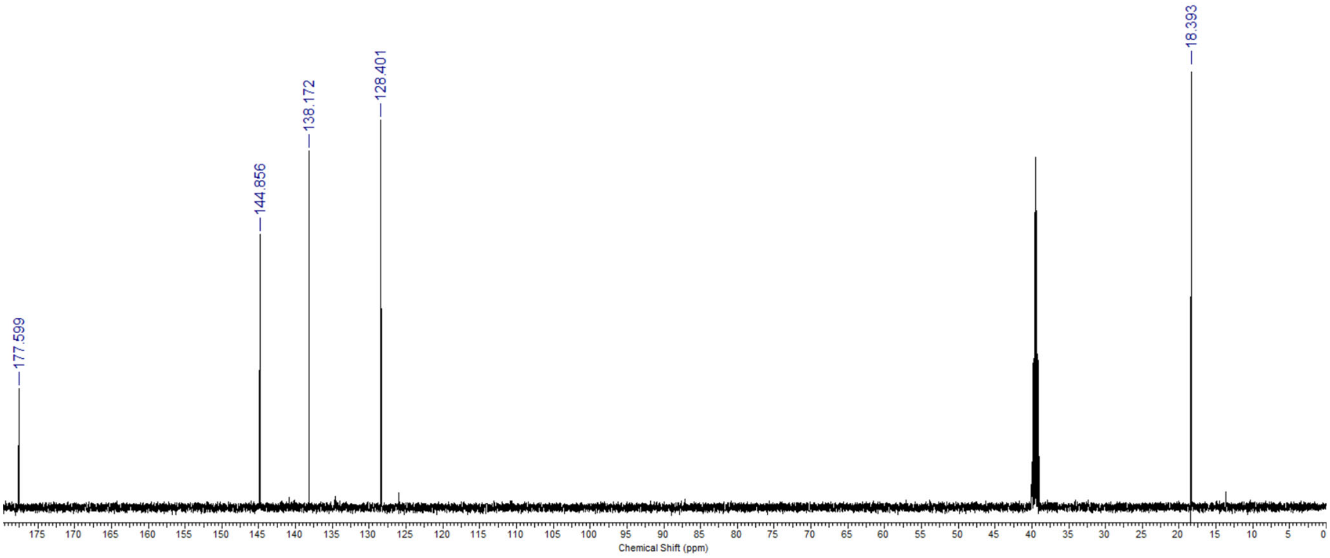

Figure S13. The ^{13}C-NMR spectrum for the compound **3a.**

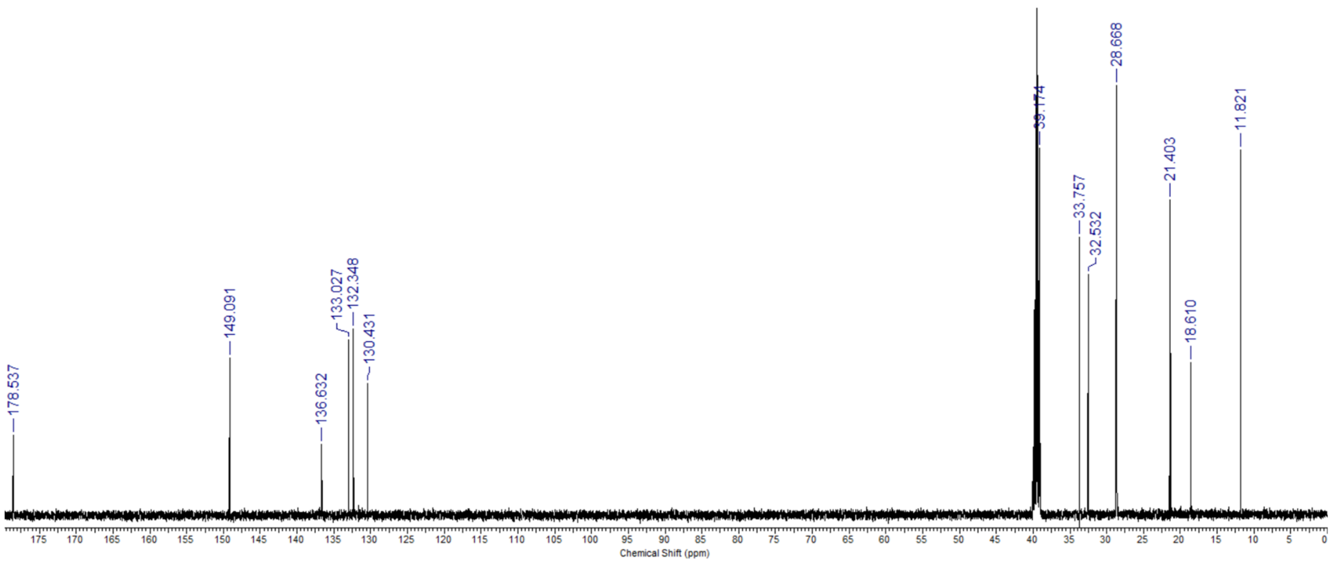

Figure S14. The ^{13}C-NMR spectrum for the compound **3b**.

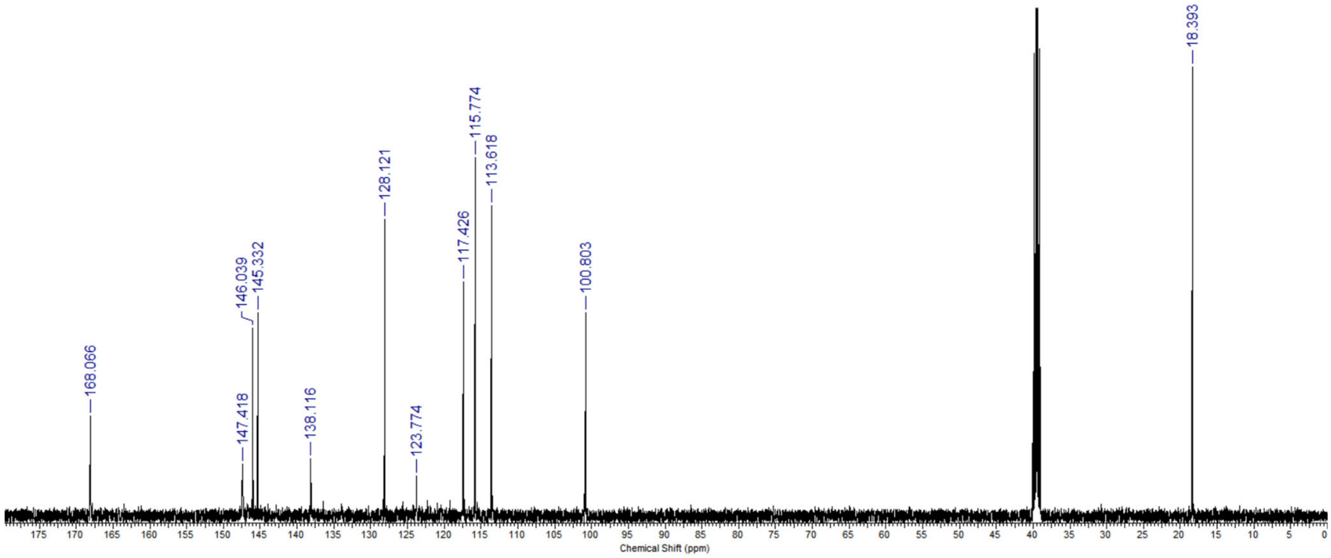

Figure S15. The ^{13}C-NMR spectrum for the compound **5a.**

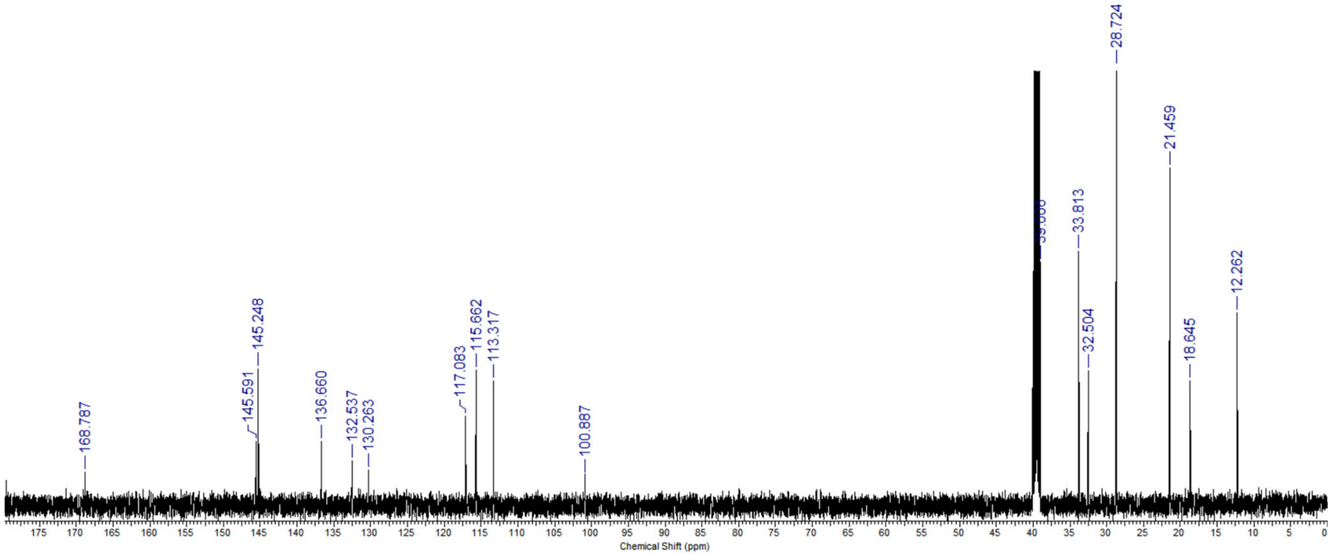

Figure S16. The ^{13}C-NMR spectrum for the compound **5b**.
